# Undergraduate setup for measuring the Bell inequalities and performing quantum state tomography

**DOI:** 10.1140/epjqt/s40507-024-00298-y

**Published:** 2024-12-19

**Authors:** Raul Lahoz Sanz, Lidia Lozano Martín, Adrià Brú i Cortés, Martí Duocastella, Jose M. Gomez, Bruno Juliá-Díaz

**Affiliations:** 1 Departament de Física Quàntica i Astrofísica, Facultat de Física, Universitat de Barcelona (UB), C. Martí i Franquès, 1, 08028 Barcelona, Spain; 2 Institut de Ciències del Cosmos (ICCUB), Universitat de Barcelona (UB), C. Martí i Franquès, 1, 08028 Barcelona, Spain; 3 Department of Applied Physics, Universitat de Barcelona (UB), C. Martí i Franquès, 1, 08028 Barcelona, Spain; 4 Departament d’Enginyeria Electrònica i Biomèdica, Universitat de Barcelona (UB), C. Martí i Franquès, 1, 08028 Barcelona, Spain; 5 Institute of Nanoscience and Nanotechnology (IN2UB), Universitat de Barcelona (UB), C. Martí i Franquès, 1, 08028 Barcelona, Spain; 6 Institut d’Estudis Espacials de Catalunya (IEEC), Edifici RDIT, Campus UPC, 08860 Castelldefels (Barcelona), Spain

**Keywords:** Entanglement, Undergraduate setups, Quantum optics, Bell inequalities, Quantum state tomography

## Abstract

The growth of quantum technologies is attracting the interest of many students eager to learn concepts such as quantum entanglement or quantum superposition. However, the non-intuitive nature of these concepts poses a challenge to understanding them. Here, we present an entangled photon system which can perform a Bell test, i.e. the CHSH inequality, and can obtain the complete tomography of the two-photon state. The proposed setup is versatile, cost-effective and allows for multiple classroom operating modes. We present two variants, both facilitating the measurement of Bell inequalities and quantum state tomography. Experimental results showcase successful manipulation of the quantum state of the photons, achieving high-fidelity entangled states and significant violations of Bell’s inequalities. Our setup’s simplicity and affordability enhances accessibility for less specialized laboratories, allowing students to familiarize themselves with quantum physics concepts.

## Introduction

Quantum superposition and entanglement are key elements in the current developments in quantum technologies [1]. However, they are elusive concepts with no classical counterpart, making them difficult to understand for undergraduate students and non-quantum experts. An important step to close this gap is through hands-on experimentation. By acquiring and analyzing data from a quantum entanglement setup, students can get acquainted with quantum mechanical concepts and grasp the non-intuitive nature of quantum physics. Still, a comprehensive description of such a system, which is easily accessible for undergraduate students and suitable to generate quality data within the time frame of laboratory sessions (a couple of hours), remains difficult to find.

Quantum entanglement, the phenomenon by which two particles become linked so that the state of one affects the state of another, regardless of the distance, is central in today’s quantum technologies. Examples include quantum computation, e.g. see Shor’s algorithm [2], quantum sensing, e.g. enhancing the LIGO detecting capability [3], and quantum communications, see the Ekert91 protocol [4]. Despite its importance, quantum entanglement has been controversial since the well-known EPR paper [5]. There, Einstein, Podolsky and Rosen argued that the quantum mechanical description of a seemingly simple system composed of two particles was most likely incomplete. They introduced the notion of hidden variables, which at the time seemed more of a philosophical idea than an empirically testable one, that would make the description of nature complete.

The situation changed drastically thanks to Bell’s article in 1964 [6]. There, he found a way of experimentally setting bounds to the existence of hidden variables. He proposed specific experiments to prove quantum mechanical predictions could not be explained with hidden variables. Since then, numerous experiments have been conducted to verify his predictions. From the pioneering experiment by John F. Clauser and Stuart Freedman [7] onward, all have supported the Copenhagen interpretation, emphasizing the intrinsic randomness of nature and ruling out the possibility of including hidden variables in the theory [8–10]. The importance of these results was clearly stated by the Nobel Prize in Physics in 2022, awarded to Alain Aspect, John F. Clauser and Anton Zeilinger “for experiments with entangled photons, establishing the violation of Bell inequalities and pioneering quantum information science” [11].

In the last two decades, numerous efforts have been made to introduce this type of experiments into the laboratory curriculum with the aim of renewing courses with more “up-to-date” experiments and to render this type of experiment accessible to undergraduate students. In the universities where hand-made setups, e.g. [12–23] and commercial setups [24], e.g. [25, 26] have been implemented there has been a notable improvement in understanding concepts pertaining to quantum physics, along with a considerably higher enthusiasm among students for such technologies [20, 27]. In our university, the proposed setup serves as an advanced quantum system in our experimental labs, as part of the Advanced Quantum Laboratory of the Master’s in Quantum Science and Technologies in Barcelona.

Compared to some other implementations of this type of experiment in laboratory syllabi that use commercial systems e.g. [25, 26], having students assembling and aligning the setup presented in this article will provide them with fundamental knowledge about optics. Knowledge that is crucial for any other work with quantum optics and can’t be acquired using pre-assembled commercial setups [24].

The main goal of this paper is to present two experimental setups for undergraduate students that allow a thorough study of the Bell inequalities. For this purpose, we describe the implementation, operation, and alignment of two such setups, enabling students to build them from scratch. Thus, our detailed guidelines offer students a pathway to their first hands-on experience with quantum concepts.

To provide a concise yet self-contained document, we describe the essential theoretical formalism needed to understand the experiment, followed by a detailed description of the practical setup implementation. The article is organized as follows. First, in Sect. 2 we present the main theoretical concepts involved, including the suitable basis states to describe the two-photon states and how to perform operations on them. This section also provides a theoretical description of the quantum state tomography (QST), necessary to fully reconstruct the state (Sect. 2.2). We then introduce the Bell inequalities, particularly the CHSH inequality [28] (Sect. 2.4). The experimental setups are described in Sect. 3, providing comprehensive descriptions of the proposed implementation. Sections 3.1 and 3.2 explain the production and measurement of photons in the two respective schemes. The way to align and run the experiment is shown in Sect. 4 while the experimental results are collected in Sect. 5. Finally, in Sect. 6, we outline the qualities of our setups and the results of our experiments. We also discuss how this work can help to bring these concepts and technologies closer to a broader and less specialized audience.

## Theory

In this section, we provide the basic theoretical tools needed to understand the proposed experiments.

### Definition of states and operators

As the photon is the quantum system of our experiments, we start by defining its quantum state. It can be described in several useful bases. The most common, the canonical basis $\{ | H\rangle , | V\rangle \}$ is formed by the vectors,

1 $$ | H\rangle = \begin{pmatrix} 1 \\ 0 \end{pmatrix} \hspace{0.8cm} \text{and} \hspace{0.8cm} | V\rangle = \begin{pmatrix} 0 \\ 1 \end{pmatrix} . $$

A set of different bases, relevant for the experiment described herein, are the Diagonal and Antidiagonal basis, the Right-handed and Left-handed basis, and the *α* rotated basis. Note that, in this description, photons consist of a two-level quantum mechanical system, known as a “qubit” in the quantum information community. Expressed in terms of $\{ | H\rangle , | V\rangle \}$, these bases can be written as

2 $$\begin{aligned}& \{ | D\rangle , | A\rangle \} = \left \{ \frac{1}{\sqrt{2}} \begin{pmatrix} 1 \\ 1 \end{pmatrix} , \frac{1}{\sqrt{2}} \begin{pmatrix} 1 \\ -1 \end{pmatrix} \right \}, \end{aligned}$$

3 $$\begin{aligned}& \{ | R\rangle , | L\rangle \} = \left \{ \frac{1}{\sqrt{2}} \begin{pmatrix} 1 \\ -i \end{pmatrix} , \frac{1}{\sqrt{2}} \begin{pmatrix} 1 \\ i \end{pmatrix} \right \}, \end{aligned}$$

4 $$\begin{aligned}& \{ | H_{\alpha}\rangle , | V_{\alpha}\rangle \} = \left \{ \begin{pmatrix} \cos \alpha \\ \sin \alpha \end{pmatrix} , \begin{pmatrix} - \sin \alpha \\ \cos \alpha \end{pmatrix} \right \}. \end{aligned}$$

**Figure 1:**
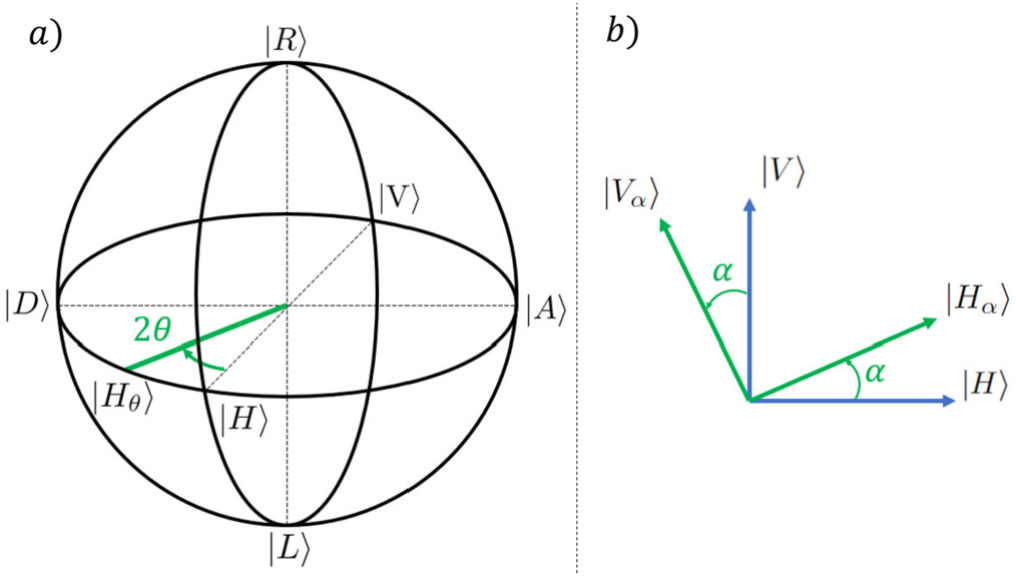
a) Visual representation of the Bloch sphere. All possible states of a single qubit are contained in the surface of the sphere. b) Representation of the $\{ | H\rangle , | V\rangle \}$ and $\{ | H_{\alpha}\rangle , | V_{\alpha}\rangle \}$ bases. The equator of the Bloch sphere is the circle generated by the $\{ | H_{\alpha}\rangle , | V_{\alpha}\rangle \}$ states

Where we define the counterclockwise direction *α* as positive when we observe the light moving away from us. Thus, in Fig. 1 b), the light propagates towards the interior of the paper.

The way to perform unitary operations on quantum states without measuring them is using retarder plates, particularly half-wave plates (HWP) and quarter-wave plates (QWP). Waveplates delay one polarization component with respect to the other due to a difference in refractive index depending on the orientation of the material, featuring a direction where the light travels faster (fast axis). As these operations are important in our setup, we provide a formal definition of their actions in the $\{| H\rangle , | V\rangle \}$ basis. The action of a HWP and a QWP with their fast axis set at an angle *θ* w.r.t the horizontal, i.e. the fast axis pointing at the direction $| H_{\theta}\rangle $, is described by

$$\begin{aligned} \mathrm{HWP}_{\theta } =& e^{-i\frac{\pi}{2}} \begin{pmatrix} \cos 2\theta & \sin 2\theta \\ \sin 2\theta & - \cos 2\theta \end{pmatrix} \,, \\ \mathrm{QWP}_{\theta } =& e^{-i\frac{\pi}{4}} \begin{pmatrix} \cos ^{2}\theta + i \sin ^{2}\theta & (1-i)\sin \theta \cos \theta \\ (1-i)\sin \theta \cos \theta & \sin ^{2}\theta + i \cos ^{2}\theta \end{pmatrix} . \end{aligned}$$

Importantly, all these operations can be represented in the Bloch sphere, as shown in Fig. 1 a) [22]. A HWP (QWP) with its fast axis set at an angle defined by the vector $| H_{\theta}\rangle $, performs rotations of 180^∘^ (90^∘^) of any single photon state with respect to the axis defined by the direction of the fast axis.

### Reconstruction of a general two-photon state. Quantum state tomography

The next step is to introduce two-photon states, which represent the minimal photonic system which can exhibit quantum entanglement. As customary in quantum optics and quantum information, we call Alice and Bob the two individuals that measure the first and the second photon, respectively.

The general case of non-pure two-photon states can be fully described by the density matrix. In other words, by experimentally measuring the density matrix, one can gather all the necessary information to assess two-photon states, a process known as quantum state tomography. The density matrix can be written as,

5 $$ \hat{\rho} = \begin{pmatrix} A_{1} & B_{1}e^{i\phi _{1}} & B_{2}e^{i\phi _{2}} & B_{3}e^{i\phi _{3}} \\ B_{1}e^{-i\phi _{1}} & A_{2} & B_{4}e^{i\phi _{4}} & B_{5}e^{i\phi _{5}} \\ B_{2}e^{-i\phi _{2}} & B_{4}e^{-i\phi _{4}} & A_{3} & B_{6}e^{i\phi _{6}} \\ B_{3}e^{-i\phi _{3}} & B_{5}e^{-i\phi _{5}} & B_{6}e^{-i\phi _{6}} & A_{4} \end{pmatrix} \,, $$

where the basis used is $\{|HH\rangle , |HV\rangle , |VH\rangle , |VV\rangle \}$. Note that this matrix is hermitian $\rho =\rho ^{\dagger}$ and thus semi-definite positive. Also, the trace has to be equal to 1, i.e. $A_{1} + A_{2} + A_{3} + A_{4} = 1$. Thus, we need $16-1=15$ parameters to fully characterize the matrix. For our experiments, it is useful to expand the density matrix as a sum of tensor products of two Pauli matrices, see for instance [22],

6 $$ \hat{\rho} = \frac{1}{4} \sum _{i,j=0}^{3} S_{ij} \cdot \hat{\sigma _{i}} \otimes \hat{\sigma _{j}}\,, $$

where $\hat{\sigma _{i}}$ are the identity and the usual Pauli matrices defined as

$$\begin{aligned} \hat{\sigma _{0}} = | V\rangle\!\langle V| + | H\rangle\!\langle H| = \begin{pmatrix} 1 & 0 \\ 0 & 1 \end{pmatrix} \,, \\ \hat{\sigma _{1}} = | D\rangle\!\langle D| - | A\rangle\!\langle A| = \begin{pmatrix} 0 & 1 \\ 1 & 0 \end{pmatrix} \,, \\ \hat{\sigma _{2}} = | L\rangle\!\langle L| - | R\rangle\!\langle R| = \begin{pmatrix} 0 & -i \\ i & 0 \end{pmatrix} \,, \\ \hat{\sigma _{3}} = | H\rangle\!\langle H| - | V\rangle\!\langle V| = \begin{pmatrix} 1 & 0 \\ 0 & -1 \end{pmatrix} . \end{aligned}$$

Importantly, the coefficients defining the state, $S_{ij}$ in Eq. (6), named Stokes coefficients, can be obtained from combined experimental measurements of the two photons in the state. For instance,

7 $$ S_{00} = P_{| HH\rangle } + P_{| HV\rangle } + P_{| VH\rangle } + P_{| VV\rangle }, $$

where $P_{| \sigma \sigma '\rangle }$ is the joint probability that Alice and Bob have of obtaining their respective photons in the states $| \sigma\rangle $ and $| \sigma '\rangle $ when Alice is measuring in the basis $\{ | \sigma\rangle , | \sigma ^{\perp}\rangle \}$ and Bob is using the $\{ | \sigma '\rangle , | \sigma ^{\prime \,\perp}\rangle \}$ basis. The explicit expressions for all Stokes coefficients can be found in Appendix B. This forms the essentials of quantum state tomography.

In our experiment we produce two-photon states which are pure states. They are a particular case of the general one in Eq. (5), and can be written as

8 $$ | \Psi\rangle = a_{0} | HH\rangle + a_{1} | HV\rangle + a_{2} | VH\rangle + a_{3} | VV\rangle , $$

with $a_{i}$ ($i=0, 1, 2, 3$) complex coefficients such that $\sum _{i=0}^{3}|a_{i}|^{2} = 1$.

To compare how similar the two distinct two-photon states are, $\hat{\rho _{1}}$ and $\hat{\rho _{2}}$, we define the fidelity, ${\mathrm{F}}(\hat{\rho _{1}}, \hat{\rho _{2}})$, [29],

9 $$ \mathrm{F}(\hat{\rho _{1}}, \hat{\rho _{2}}) = \left (\mathrm{Tr} \left [ \sqrt{ \sqrt{\hat{\rho _{1}}}\hat{\rho _{2}}\sqrt{\hat{\rho _{1}}}} \right ] \right )^{2}. $$

Furthermore, if one of the two states under comparison is pure ($\hat{\rho _{2}} = | \Psi _{2}\rangle\!\langle \Psi _{2}|$), the expression defined in Eq. (9) becomes

10 $$ \mathrm{F}(\hat{\rho _{1}}, \hat{\rho _{2}}) = \mathrm{Tr}(\hat{\rho _{1}} | \Psi _{2}\rangle\!\langle \Psi _{2}|) = \langle \Psi _{2}|\hat{\rho _{1}} | \Psi _{2}\rangle . $$

The values of the fidelity fall between 0 and 1. Fidelity 1 is only achieved if both states are equal, while fidelity 0 is obtained for orthogonal states.

### Entangled states

A key concept for this work is that of quantum entanglement. Working with pure states of the form of Eq. (8), a two-photon state is said to be entangled if it cannot be written as a separable state,

11 $$ | \Psi\rangle _{\mathrm{Separable}} = | \psi\rangle \otimes | \varphi\rangle . $$

with $| \psi\rangle $ and $| \varphi\rangle $ single photon states. Note that, in a separable state, the outcomes of the measurements of Alice and Bob are completely independent, while in entangled states, quantum correlations arise between the two outcomes.

A set of well-known and useful entangled states are the so-called Bell states,

12 $$\begin{aligned} | \Phi ^{+}\rangle &= {\frac{1}{\sqrt{2}}} (| HH\rangle + | VV\rangle ), \\ | \Phi ^{-} \rangle &= {\frac{1}{\sqrt{2}}} (| HH\rangle - | VV\rangle ), \\ | \Psi ^{+}\rangle &= {\frac{1}{\sqrt{2}}} (| HV\rangle + | VH\rangle ), \\ | \Psi ^{-}\rangle &= {\frac{1}{\sqrt{2}}} (| HV\rangle - | VH\rangle ). \end{aligned}$$

These states, in turn, form a basis of the two-photon Hilbert space.

### Bell test — CHSH inequality

The correlations stemming from the non-separability of states rose important criticism. Notably, in the so-called EPR paradox stated in Ref. [5], it was argued that the description of nature is probably incomplete, calling for the existence of so-called hidden variables. John Bell [6] introduced the first empirical approach to distinguish predictions from hidden variable theories. Since then, a series of Bell-type inequalities (i.e. Bell tests) have been developed to check if the quantum state associated to two particles follows a non-local behavior. In particular, quantum mechanics produces predictions which violate Bell inequalities (they are incompatible with hidden variable theories). In our case, we consider the CHSH inequality [28], which was the one used in the pioneering article by Aspect and collaborators [8], and also in the pedagogical setup of Ref.-[18].

**Figure 2:**
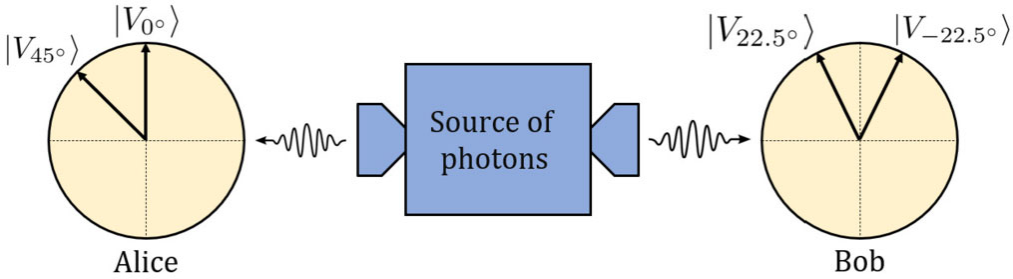
Sketch of the CHSH protocol: A source generates photon pairs always in the same state, sending one to Alice and the other to Bob. They can determine if the received pairs are entangled, performing measurements in different bases and sharing their results

To carry out the Bell test, we consider the scenario described in Fig. 2. There, a source generates pairs of photons, named signal and idler, that are always produced in the same manner, and thus in the same quantum state, and that are sent in different directions. The receivers of these photons, again Alice and Bob, can determine whether the pairs of photons they share are entangled or not by performing measurements of the individual photons separately and communicating the results.

We define the functions $\mathrm{{a}(\alpha )}$ ($\mathrm{{b}(\beta )}$) as $\mathrm{{a}(\alpha ) = 1}$ ($\mathrm{{b}(\beta ) = 1}$) if Alice (Bob) measures the signal (idler) photon in the state $| V_{\alpha}\rangle $ and $\mathrm{{a}(\alpha ) = }-\mathrm{1}$ ($\mathrm{{b}(\beta ) = }-\mathrm{1}$) if Alice (Bob) measures the signal (idler) photon in the state $| H_{\alpha}\rangle $. Then, we define the correlation function $E(\alpha , \beta )$, i.e. the average of the product of both measurements, as

13 $$ \begin{aligned} &E(\alpha , \beta ) = \langle \mathrm{{a}(\alpha ) }\cdot \mathrm{{b}( \beta ) \rangle = }\\ &= P_{| V_{\alpha }V_{\beta}\rangle } - P_{| V_{\alpha }H_{\beta}\rangle } - P_{ | H_{\alpha }V_{\beta}\rangle } + P_{| H_{\alpha }H_{\beta}\rangle }. \end{aligned} $$

In the CHSH inequality, Alice (Bob) measures the state of the photons in two different states, $\alpha = 0^{\circ}$ ($\beta = 22.5^{\circ}$) and $\alpha ' = 45^{\circ}$ ($\beta ' = -22.5^{\circ}$). Thus, Alice and Bob obtain four different values of Eq. (13); one for each combination of angles. $E(\alpha , \beta )$, $E(\alpha ', \beta )$, $E(\alpha , \beta ')$ and $E(\alpha , \beta ')$. Using these four values, we define the functions *S* and $S'$ as:

14 $$\begin{aligned}& S = E(\alpha , \beta ) + E(\alpha , \beta ') + E(\alpha ', \beta ) - E( \alpha ', \beta '), \end{aligned}$$

15 $$\begin{aligned}& S' = E(\alpha , \beta ) + E(\alpha , \beta ') - E(\alpha ', \beta ) + E( \alpha ', \beta ') . \end{aligned}$$

These functions are constructed to always yield a value between −2 and +2 when working with classical correlations, including the case of hidden-variable theories. In contrast, their value falls between $-2\sqrt{2}$ and $+2\sqrt{2}$ when we compute the averages with quantum mechanics. Specifically, for each Bell state, one of them yields a result of zero, while the other provides a value equal to $-2\sqrt{2}$ or $+2\sqrt{2}$:

- If the two photons are in the state $| \Phi ^{+(-)}\rangle $, we obtain $\langle S \rangle = 2\sqrt{2}(0)$ and $\langle S' \rangle = 0(2\sqrt{2})$.
- If the two photons are in the state $| \Psi ^{+(-)}\rangle $, we obtain $\langle S \rangle = 0(-2\sqrt{2})$ and $\langle S' \rangle = -2\sqrt{2}(0)$.

The fact that different Bell states require different Bell test functions *S* and $S'$ is often not emphasized. In our case, we can precisely control the relative phase between components in the wave function of photon pairs along with the use of a HWP in one of the photons’ paths. Thus, with our setups, we have the ability to generate the four maximally entangled Bell states, as is described later in Sect. 3.1. These two features set our work apart from other pedagogical setups.

## Experimental setups

The two setups presented herein consist of a photon pair production part followed by a photon detection part. The photon pair production was based on spontaneous parametric down conversion in two type I BBO crystals, firstly proposed and accomplished experimentally in Ref. [30] and adapted to the undergraduate laboratory in Refs. [18, 19]. The details of similar experiments have been described in later Refs. [20, 21].

Both setups enable performing a full two-photon state tomography and a Bell test, and incorporate improvements at both the technical and conceptual level with respect to previous works. Among them, our setups allow us to prepare different Bell states, which emphasizes the fact that Bell tests are tailored for specific states. Also, both options feature a significantly simpler optical alignment of the elements of the setup and are fairly robust. Importantly, the measurement time is very reasonable: a Bell test can be performed in less than one hour, providing ample options for lab experimentation at both the undergraduate and master’s level.

**Figure 3:**
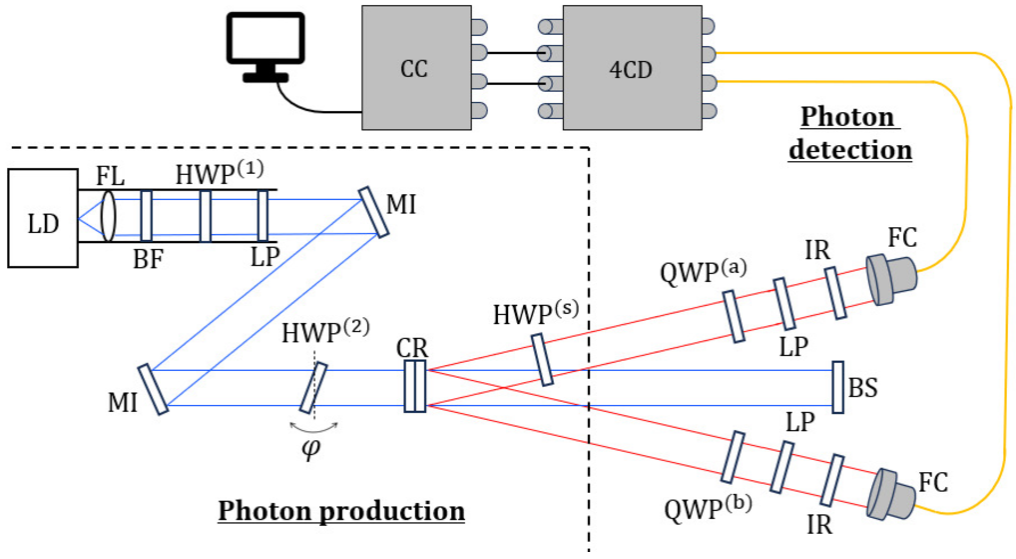
Scheme setup 1. LD. laser diode, FL. focusing lens, BF. blue filter, LP. linear polarizer, MI. mirror, CR. BBO crystals, HWP. half-wave plate, QWP. quarter-wave plate, IR. infrared filter, FC. fiber-coupler lenses, 4CD. 4-channel detector, CC. Coincidence Circuit

**Figure 4:**
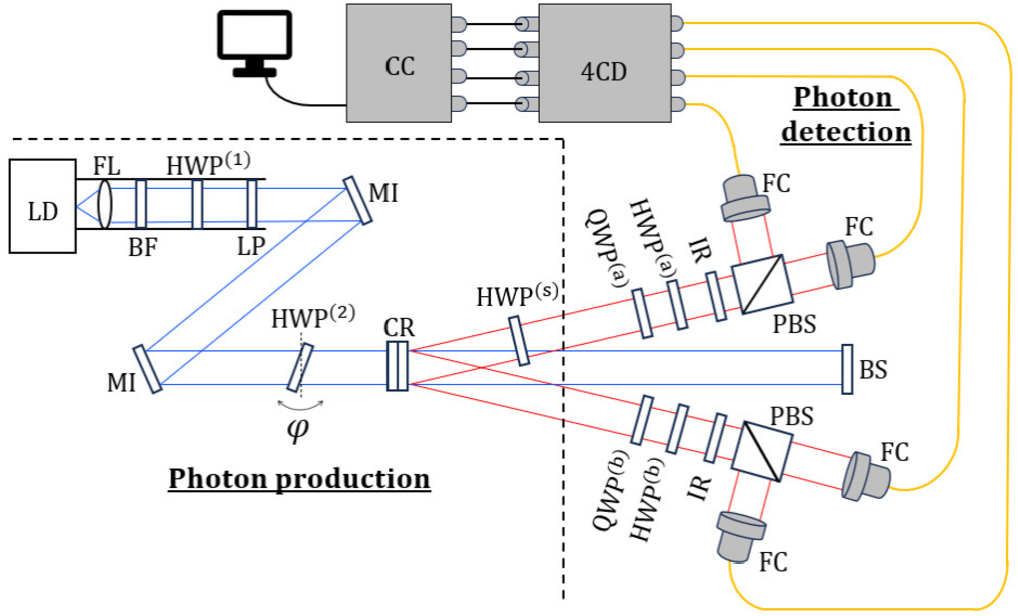
Scheme setup 2. PBS. polarizing beam splitter. The rest of the elements are labeled in the same way as in Fig. 3

A detailed list of the necessary equipment for each setup is compiled in Appendix A. The main difference between the two setups lies in the photon detection process. The first setup, illustrated in Fig. 3, employs only two inputs of the 4-channel detector (SPCM-AQ4C, Excelitas Technologies) and measures the polarization of the light using a QWP and a polarizer. While simpler in terms of optical elements, this option is slower for measurements. It can only provide the number of coincident photon counts passing through both polarizers in a single measurement. That is, pairs of photons that pass through both polarizers without being stopped and are detected simultaneously. The second setup, depicted in Fig. 4, needs all 4 inputs of the detector and directs photons to different detectors based on their polarization using polarizing beam splitters (PBS). Although this option requires more optical elements, it is faster for measurements as it allows for the measurement of photon counts in any of the states of a given basis in a single measurement.

In both of our setups, as in some other pedagogical experiments [13, 14, 17, 20], collimating lenses (F810FC-780, ThorLabs) or microscope objectives and optical fibers are used to capture photons. This offers a significant advantage in alignment, as is discussed in Sect. 4.1. In some previous works [12, 18, 19] the alignment of the system and the capture of photons was performed without the use of optical fibers. To count coincidences between two channels, we have replicated the circuit described in Ref. [19] modifying the capacitors to reduce the coincidence window to 90 ns and adding USB connectivity.

### Photon production

As shown in Fig. 3 and Fig. 4, the two-photon production part is similar in both setups. It also shares many elements with previous works, in particular with that of Dehlinger and Mitchell [18, 19]. In more detail, we use a 405 nm laser beam (L404P400M, ThorLabs) working at 400 mW that emits horizontally-polarized light.

$$ | \Psi\rangle = | H\rangle _{\mathrm{{Pump}}} . $$

To switch from horizontal to diagonal light with almost no energy loss, we employ a HWP with its optical axis set at an angle $\theta = 22.5^{\circ}$

$$ \mathrm{HWP}^{(1)}_{\theta = 22.5^{\circ}} | H\rangle = e^{i \cdot \frac{\pi}{2}} | D\rangle = | D\rangle . $$

Additionally, we place a polarizer set at a 45^∘^ angle to further ensure the desired polarization sate. Thus, the quantum state after the polarizer reads,

$$ | \Psi\rangle = | D\rangle _{\mathrm{{Pump}}}. $$

Afterwards, the light gets reflected by the two 3D-precision mirrors and passes through a HWP with its fast axis parallel to the optical table. This HWP is mounted on a goniometer (RP01/M, ThorLabs) that allows us to tilt it around the axis perpendicular to the optical table. With this tilt angle, *φ* we can vary the relative phase ($\phi ( \varphi )$) between the $| H\rangle $ and $| V\rangle $ component.

16 $$ | \Psi\rangle = \frac{1}{\sqrt{2}}\left ( | H\rangle _{\mathrm{{Pump}}} + e^{i \cdot \phi (\varphi )} | V\rangle _{\mathrm{{Pump}}} \right ). $$

Note that, at this stage, we have produced a photon in a superposition of both horizontal and vertical polarization. To generate entangled photons, we exploit a phenomenon called spontaneous parametric down-conversion (SPDC) Ref. [30]. To this end, we place a pair of barium borate (BBO) crystals (both Type I, cut at a phase-matching angle $\theta =29.2^{\circ}$, with dimensions $6\times 6\times 0.1\text{ mm}$, optically contacted on, and each one rotated 90^∘^ with respect to the other) in the light path. Upon interaction with the BBO crystals, an initial single photon, called pump, can generate two down-converted (and thus, less energetic) photons. Although the probability of this process is low (one in a million, at best), the high photon flux that reaches the BBO crystal ensures repeatable generation of Bell states.

**Figure 5:**
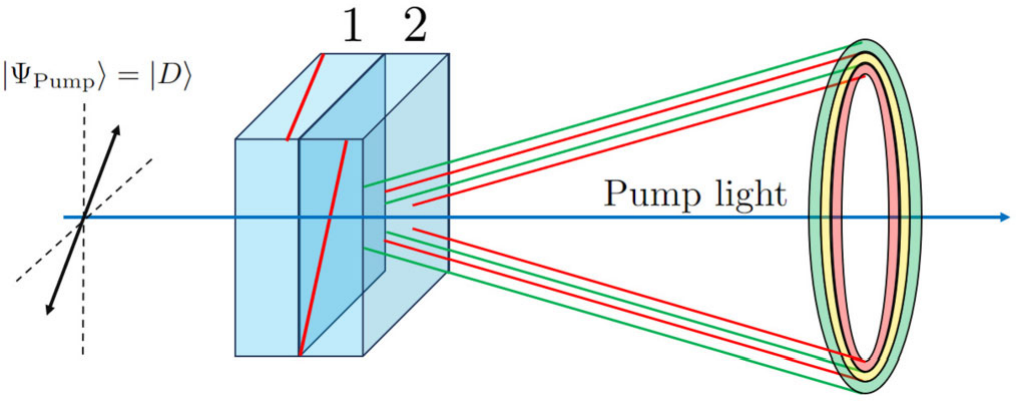
Scheme of the production of entangled photons using two Type-I BBO crystals. The optical axes of the crystals represented as red line are forming 90^∘^

The plane formed by the optical axis of the BBO crystal and the direction of propagation of the incident pump photon is known as the SPDC plane. Only a pump photon with polarization contained in the SPDC plane can experience SPDC and generate two photons. In this case, both photons feature a perpendicular polarization with respect to that of the incident pump photon. Instead, if the polarization of the pump photon is perpendicular to the BBO plane, the BBO crystal does not produce pairs of photons [30]. Thus, by illuminating the first (second) BBO crystal with horizontal (vertical) light, pairs of photons can be produced, both with vertical (horizontal) polarization, as shown in Fig. 5. The green and red cones are the *V*-polarized and *H*-polarized light cones, respectively.

As the photons produced in the first BBO crystal have extraordinary polarization in the second BBO crystal, then, a relative phase $\phi _{\mathrm{{BBO}}}$ appears between the pair of photons produced in the first and the second crystal.

17 $$\begin{aligned} | H\rangle _{\mathrm{{Pump}}} &\xrightarrow{\mathrm{BBO's}} | V\rangle _{\mathrm{{s}}}\otimes | V\rangle _{\mathrm{{i}}} , \end{aligned}$$

18 $$\begin{aligned} | V\rangle _{\mathrm{{Pump}}} &\xrightarrow{\mathrm{BBO's}} e^{i \cdot \phi _{\mathrm{{BBO}}}} \cdot | H\rangle _{\mathrm{{s}}}\otimes | H\rangle _{\mathrm{{i}}}. \end{aligned}$$

In our experiments, we excite the BBO crystals with diagonally-polarized pumped light, that is, light in an equal superposition between the $| H\rangle $ and $| V\rangle $ states. These photons can be down-converted in both crystals. Thus, in the region of space where both light cones overlap (yellow region in Fig. 5) the photons that we receive are indistinguishable, i.e. we cannot tell in which BBO crystal they were generated. What we do know is that, if we measure the polarization of one of them, the polarization of the other one is the same. It is precisely this indistinguishability between two-photon paths what gives rise to the entanglement.

Let us consider the pair of BBO crystals with their optical axes pointing in the vertical and horizontal direction. When one pump photon in the state Eq. (16) goes through them and suffers SPDC, following Eqs. (17) and (18) produces

19 $$ | \Psi _{\mathrm{{EPR}}}\rangle = \frac{1}{\sqrt{2}}\left ( | VV\rangle + e^{i \cdot (\phi (\varphi )+\phi _{\mathrm{{BBO}}})} | HH\rangle \right ). $$

Changing the tilt angle *φ* of the $\mathrm{{HWP}^{(2)}}$, we can control the relative phase between the $| VV\rangle $ and the $| HH\rangle $ components. Let us call $\varphi ^{+}$ and $\varphi ^{-}$ the angles for which we obtain 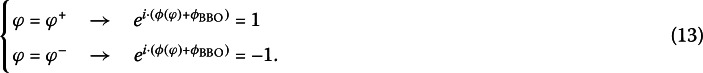


Finally, the four Bell states can be produced with this setup by adding a HWP. In particular, a HWP ($\mathrm{{HWP}^{(s)}}$) placed in the optical path corresponding to the signal photons, enables obtaining all four Bell states. As shown in Table 1, these states depend on the angles *φ* and $\theta _{s}$, where $\theta _{s}$ is the angle that forms the fast axis of the $\mathrm{{HWP}^{(s)}}$ with respect to the horizontal direction.

**Table 1:** Value of the variables *φ* and $\theta _{s}$ for the production of all four Bell States

Bell state	Angle $\theta _{s}$	Angle *φ*
$| \Phi ^{+}\rangle = \frac{1}{\sqrt{2}} \left ( | HH\rangle + | VV\rangle \right )$	0^∘^	$\varphi = \varphi ^{+}$
$| \Phi ^{-}\rangle = \frac{1}{\sqrt{2}} \left ( | HH\rangle - | VV\rangle \right )$	0^∘^	$\varphi = \varphi ^{-}$
$| \Psi ^{+}\rangle = \frac{1}{\sqrt{2}} \left ( | HV\rangle + | VH\rangle \right )$	45^∘^	$\varphi = \varphi ^{+}$
$| \Psi ^{-}\rangle = \frac{1}{\sqrt{2}} \left ( | HV\rangle - | VH\rangle \right )$	45^∘^	$\varphi = \varphi ^{-}$

### Photon detection

The key distinction between both setups lies in the photon detection part. In particular, in the following three aspects: 1) the number of detector channels employed, 2) the way we perform unitary transformations on the photon individually and, 3) the way the polarization is measured.

The state of one photon can be expressed in any of the bases introduced previously,

20 $$ \begin{aligned} | \Psi\rangle &= C_{V} | V\rangle + C_{H} | H\rangle \\ &= C_{V_{\alpha}} | V_{\alpha}\rangle + C_{H_{\alpha}} | H_{\alpha}\rangle \\ &= C_{R} | R\rangle + C_{L} | L\rangle , \end{aligned} $$

where $C_{i}$ are complex numbers. The squared modulus of these coefficients represents the probability of finding the photon in that state. Our main objective is to measure the number of photons that reach our detectors in each state of a given basis. However, we are limited in the information that we can gather. For example, by varying the polarizer angle *α* with respect to the vertical direction in the setup shown in Fig. 3, we are restricted to measure the states located on the equator of the Bloch sphere $\{ | V_{\alpha}\rangle , | H_{\alpha}\rangle \}$ (Fig. 1 a). The setup depicted in Fig. 4 is even more restrictive, allowing access to only the basis $\{ | V\rangle , | H\rangle \}$. To measure photons in the various bases of interest in each setup, which is needed for the Bell test and quantum state tomography experiments, retarder plates are required. A brief guideline on how to use them is presented in the following subsections.

#### Measurements in setup 1

**Figure 6:**
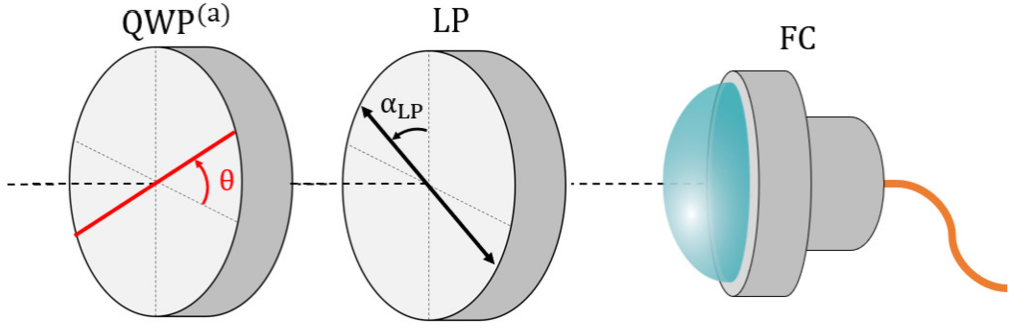
Measurement scheme in the signal photons’ arm in Setup 1. Photons travel from left to right, first passing through the QWP with its fast axis (red line) set at an angle *θ* with respect to the horizontal. They then go through the linear polarizer, oriented at an angle $\alpha _{\mathrm{LP}}$ relative to the vertical. In the idler photons’ arm, the scheme is the same

In the first setup, to measure the photons polarization we need a QWP and a linear polarizer, as depicted in Fig. 6. By placing the QWP at $\theta = \alpha $,

$$ \begin{aligned} &\mathrm{{QWP}^{(a)}_{\theta = \alpha}(| H_{\alpha}\rangle ) = | H_{\alpha}\rangle }, \\ &\mathrm{{QWP}^{(a)}_{\theta = \alpha}(| V_{\alpha}\rangle ) = | V_{\alpha}\rangle }, \end{aligned} $$

the QWP acts as an identity operator for the states $| V_{\alpha}\rangle $ and $| H_{\alpha}\rangle $. In other words, the QWP does not alter the photon state. Thus, the number of photons in the $| V_{\alpha}\rangle $ and $| H_{\alpha}\rangle $ states can be measured by simply placing the polarizer at an angle $\alpha _{\mathrm{LP}} = \alpha $ and $\alpha _{\mathrm{LP}} = \alpha + 90^{\circ}$ respectively. Instead, by placing the QWP at $\theta = 45^{\circ}$,

$$\begin{aligned} &\mathrm{{QWP}^{(a)}_{\theta = 45^{\circ}}(| L\rangle ) = | H\rangle }, \\ &\mathrm{{QWP}^{(a)}_{\theta = 45^{\circ}}(| R\rangle ) = | V\rangle }, \end{aligned}$$

the entire $| R\rangle (| L\rangle )$ component of our state described in the Eq. (21) becomes $| V\rangle (| H\rangle )$. Therefore, by placing the polarizer at an angle $\alpha _{\mathrm{LP}} = 0^{\circ}$ ($\alpha _{\mathrm{LP}} = 90^{ \circ}$), the statistics corresponding to the photon state $| R\rangle $ ($| L\rangle $) can be directly accessed. A summary of the procedure to measure the statistics for the different photon states in setup 1 is shown in Table 2.

**Table 2:** Value of the angles of the QWP and the linear polarizer for obtaining all different photon-state statistics using the setup depicted in Fig. 3

Angle QWP	Angle LP	Counts detected
*α*	*α*	$N_{| V_{\alpha}\rangle }$
*α*	*α* + 90^∘^	$N_{| H_{\alpha}\rangle }$
45^∘^	0^∘^	$N_{| R\rangle }$
45^∘^	90^∘^	$N_{| L\rangle }$

#### Measurements in setup 2

**Figure 7:**
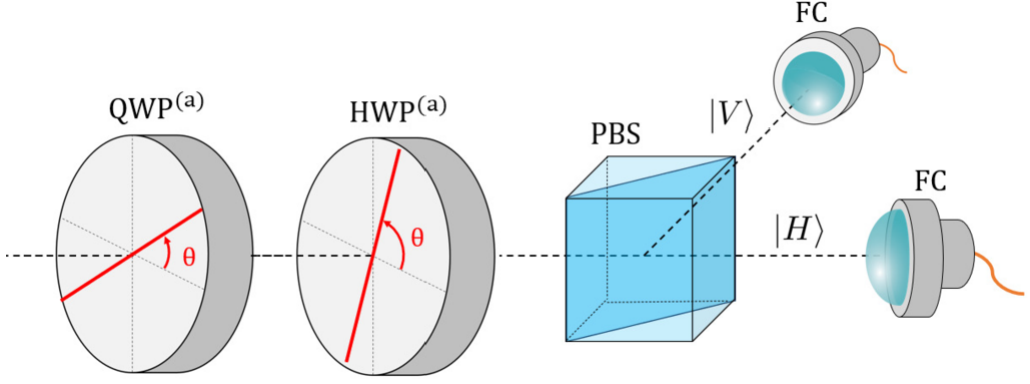
Measurement scheme in the signal photons’ arm in Setup 2. Photons travel from left to right, first passing through the QWP and HWP before going through the polarizing beam splitter (PBS). In the PBS, photons with vertical polarization get reflected and photons with horizontal polarization gets transmitted

In the second setup, we measure the photon polarization using a QWP, followed by a HWP and a polarizing beam splitter (PBS), as depicted in Fig. 7. Thus, photons with vertical (horizontal) polarization are collected by the fiber-coupling lens placed in the reflected (transmitted) path of the PBS. By placing the QWP at $\theta = \alpha $ and the HWP at $\theta = \frac{\alpha}{2}$, the following relationships hold

$$ \begin{aligned} &\mathrm{{HWP}^{(a)}_{\theta = \frac{\alpha}{2}}(}\mathrm{{QWP}^{(a)}_{ \theta = \alpha}(| H_{\alpha}\rangle )) = }\mathrm{{HWP}^{(a)}_{\theta = \frac{\alpha}{2}}(| H_{\alpha}\rangle ) = | H\rangle }, \\ &\mathrm{{HWP}^{(a)}_{\theta = \frac{\alpha}{2}}(}\mathrm{{QWP}^{(a)}_{\theta = \alpha}(| V_{\alpha}\rangle )) = }\mathrm{{HWP}^{(a)}_{\theta = \frac{\alpha}{2}}(| V_{\alpha}\rangle ) = | V\rangle }, \end{aligned} $$

where the $| V_{\alpha}\rangle $ and $| H_{\alpha}\rangle $ components of any arbitrary state described in Eq. (21) are transformed into $| V\rangle $ and $| H\rangle $ components, respectively. When these photons pass through the PBS, in the reflected (transmitted) path, the vertically (horizontally) polarized photons follow the statistics of photons in the state $| V_{\alpha}\rangle $ ($| H_{\alpha}\rangle $). On the other hand, if we place the QWP at $\theta = 45^{\circ}$ and the HWP at $\theta = 0^{\circ}$, we obtain

$$ \begin{aligned} &\mathrm{{HWP}^{(a)}_{\theta = 0^{\circ}}(}\mathrm{{QWP}^{(a)}_{ \theta = 45^{\circ}}(| L\rangle )) = }\mathrm{{HWP}^{(a)}_{\theta = 0^{\circ}}( | H\rangle ) = | H\rangle }, \\ &\mathrm{{HWP}^{(a)}_{\theta = 0^{\circ}}(}\mathrm{{QWP}^{(a)}_{\theta =45^{ \circ}}(| R\rangle )) = }\mathrm{{HWP}^{(a)}_{\theta = 0^{\circ}}(| V\rangle ) = | V\rangle }, \end{aligned} $$

the entire $| R\rangle (| L\rangle )$ component of our state becomes $| V\rangle (| H\rangle )$. Therefore, in the reflected (transmitted) path of the PBS, the counting statistics of photons in the states $| R\rangle $ and $| L\rangle $ can be measured, respectively. A summary of the procedure to obtain all different photon state statistic using setup 2 is shown in Table 3.

**Table 3:** Value of the angles of the QWP and the HWP for obtaining all different photon state statistics in the reflected and transmitted path of the PBS, using the setup depicted in Fig. 4

Angle QWP	Angle HWP	Counts detected in the refelected (transmitted) path
*α*		$N_{| V_{\alpha}\rangle }$ ($N_{| H_{\alpha}\rangle }$)
45^∘^	0^∘^	$N_{| R\rangle }$ ($N_{| L\rangle }$)

## Alignment of the setup

As with any system based on single photon detection, the alignment of the different optical elements is key for retrieving sound statistics. In this section, we explain in full detail how to align our experimental setup.

### Alignment of the pump laser and detectors

Once all optical elements are assembled, it is necessary to check that the pump laser travels parallel to the optical table and through the center of the elements. This can be controlled by precisely adjusting the two mirrors (KS1, ThorLabs) where the pump beam is reflected.

To ensure that the fiber-coupling lenses (F810FC-780, ThorLabs) are aligned with respect to the BBO crystals, we use the following procedure. First, we insert the light from a low-power visible laser to the end of the fiber - the end where the photodetectors would be connected. Secondly, we check the location of the two generated laser spots on the BBO crystal. Thirdly, by using the precision mount (KS1, ThorLabs) where the fiber-coupling lenses are assembled, we adjust the position of the spots to lie at the BBO crystals. This three-step process grants that the collected light by the photodetectors provided from the BBO crystal.

### Optimal position for the rail angles

The angles of the detectors rails (and therefore the fiber-coupler lenses) determine the number of collected photons, and therefore, the statistical robustness of the experiments. Thus, it is necessary to determine the angle for which the maximum number of coincident counts is detected. Note that coincident counts from the BBO crystals can be detected when their number deviates by at least an order of magnitude compared to accidental counts ($N_{acc}$), that is, counts expected by mere chance. These accidental counts depend on the number of counts detected individually by each of the detectors ($N_{a}$ and $N_{b}$), as well as the measurement duration (T) and the coincidence window (*τ*), according to the following equation

21 $$ N_{acc} = \frac{N_{a} \cdot N_{b} \cdot \tau}{\mathrm{T}}. $$

**Figure 8:**
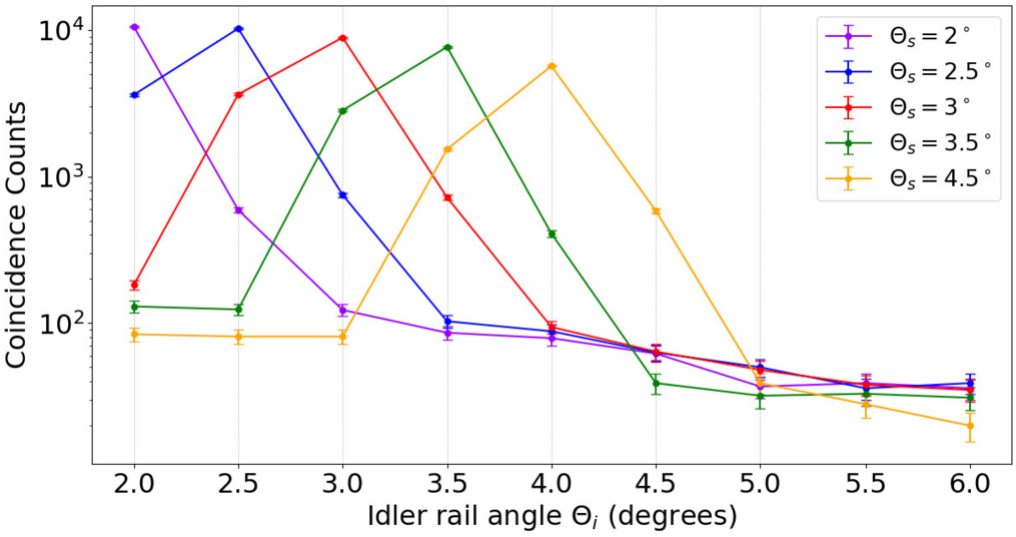
Coincidence counts and averaged accidental counts for different angular positions of the metallic rails. Each of the measurements takes 30 seconds

In our case, the value for the coincidence window is fixed and equal to 90 ns. To find the optimal position of the rails, we conduct a study in which, for a certain angle of the signal rail ($\Theta _{s}$), we record the number of detected coincidences for various positions of the idler rail ($\Theta _{i}$), as shown in Fig. 8. Interestingly, the maximum number of coincidence counts for each condition analyzed occurs when one detector is approximately at the same angle as the other detector. In addition, these local maxima slightly decreases with ($\Theta _{i}$). Note that, given the footprint of the metal rails, we cannot position both detectors at less than 2.5^∘^. Therefore, fixing the detectors at an angle $\Theta _{s} = \Theta _{i} = 2.5^{\circ}$ with respect to the pump laser beam provides the largest number of coincidence counts. This is the condition used for all the remaining measurements. Notably, at these angles, the number of coincidence counts differs by at least two orders of magnitude compared to the number of accidental counts. This indicates that the coincidence counts we detect come from photon pairs produced in the BBO crystals. It is also worth mentioning that the number of dark counts of our detector was around 350 counts per channel per second.

### Optimal position of the BBO crystals. Finding the direction of the optical axes

By design, the two BBO crystals are orthogonally oriented, as an assembly, and mounted on a rotation mount. They produce photons when the polarization of the incident light is contained in the SPDC plane of the crystal [30]. Thus, by exciting the BBO crystals with horizontally polarized pump light and rotating the BBO crystals, we can determine the four angles at which only photons in the state $| VV\rangle $ are produced. Note that at these angles the SPDC plane of one BBO crystal is parallel to the pump light polarization, while the SPDC plance of the second one is perpendicular to it. In this case, the first crystal generates photon paris in the state $| VV\rangle $ while the second one does not emit any light

**Figure 9:**
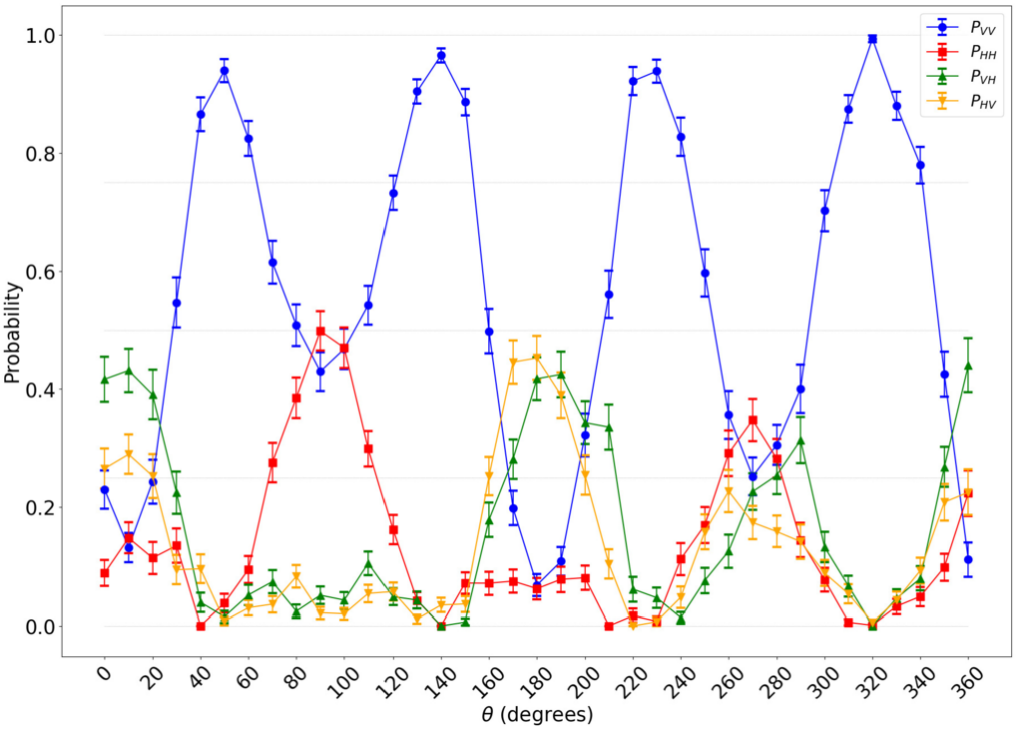
Coincidence probabilities measured for various BBO crystal angles and photon-pair states. Each measurement lasts 30 seconds

By rotating the BBO crystals in steps of 10^∘^, and measuring the photons in the states $| VV\rangle $, $| VH\rangle $, $| HV\rangle $ and $| HH\rangle $ for each angle, we obtain the dependence shown in Fig. 9. The maximal signal of photons in the $| VV\rangle $ state is found for angles 45^∘^, 135^∘^, 225^∘^ and 315^∘^. These are the optimal angles for the production of entangled photons when shining the crystals with diagonally-polarized light. In this case, one of the crystals has the optical axis pointing in the $| H\rangle $ direction and the other in the $| V\rangle $ direction.

### Finding the optimal phase-matching angle

Once the optical axes of the BBO crystals point in the $| V\rangle $ and $| H\rangle $ directions, we can further enhance the number of detected photons under illumination with diagonally polarized light. This can be achieved by finding the phase-matching condition for each crystal, that is, by aligning the phase of the light waves within the crystal to maximize their interaction.

To this end, we tilt the BBO crystals around the vertical and horizontal axis, effectively changing the angle between the propagation direction of the incidence pump photons and the plane of the BBO crystals. Note that our BBO crystals are cut at a phase-matching angle of 29.2^∘^, which is not optimized for the wavelength that we are working with (405 nm) [31, 32]. For a 405 nm wavelength, an optimal phase matching angle is $\theta =29^{\circ}$. In any case, we can maximize the number of detected photons by using the precision mount in which the crystals are placed (KS1RS, ThorLabs), as shown in Fig. 5. We achieve so despite the crystals being held together. Given that the first (second) crystal has its optical axis contained in the horizontal (vertical) plane, tilting the set of crystals around the vertical (horizontal) axis only affects the phase-matching angle of first (second) crystal.

As we vary the angle of the crystal whose optical axis is pointing in the vertical (horizontal) direction, we measure the photons reaching the detectors in the $| HH\rangle $ ($| VV\rangle $) states. The angle at which the highest number of photons is detected corresponds to the optimal phase-matching angle.

### Finding the relative phase-shift dependence with the tilt angle of the HWP^(2)^

The $\mathrm{{HWP}^{(2)}}$ has its fast axis pointing in the $| H\rangle $ direction, but is placed in a mount that allows to tilt this retarder plate around an axis perpendicular to the optical table (RP01/M, ThorLabs). This permits us to adjust the relative phase between the $| VV\rangle $ and the $| HH\rangle $ photons produced in the BBO crystals, as is described in Eq. (19).

If we measure the photons produced by the crystals in the state $| D\rangle _{s} \otimes | D\rangle _{i}$ while we vary the tilt angle *φ* of the $\mathrm{{HWP}^{(2)}}$, we expect to find the number of coincident counts following the dependence

22 $$ \begin{aligned} N_{| DD\rangle }(\varphi ) &\propto | \langle DD | \Psi _{\mathrm{{EPR}}}\rangle |^{2} \\ &\propto \frac{1}{4} \cdot (1+\cos \phi '), \end{aligned} $$

where $| \Psi _{\mathrm{EPR}}\rangle $ is the state defined in Eq. (19) and $\phi ' = \phi (\varphi )+\phi _{\mathrm{{BBO}}}$. When $\varphi = \varphi ^{+}+2\pi n$ ($\varphi = \varphi ^{-}+2\pi n$), with $n \in \mathbb{Z}$ we expect to find a maximum (minimum) in the number of pairs of photons in the state $| DD\rangle $ [30].

**Figure 10:**
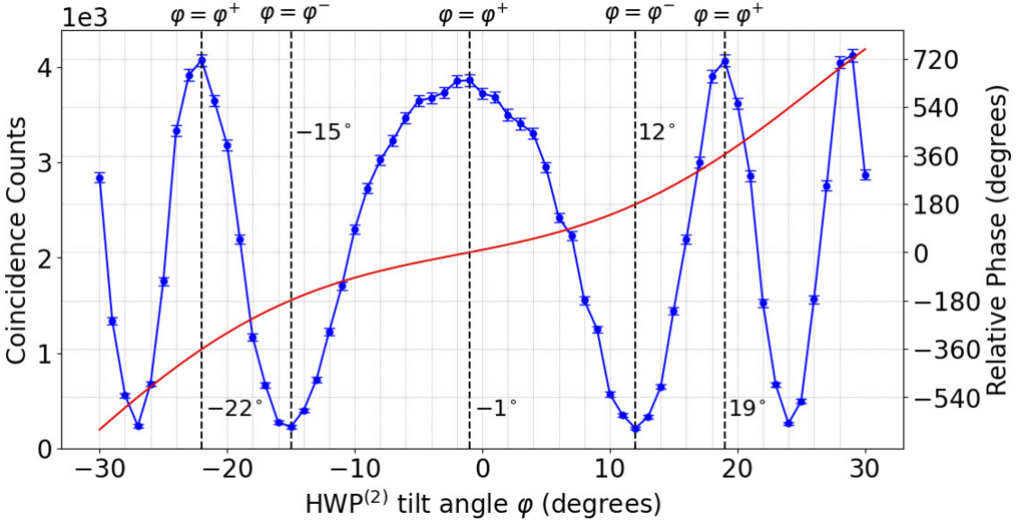
The dependence of coincidence counts, represented by blue dots, and relative phase shift, depicted as a red solid line, on the tilt angle *φ* of the $\mathrm{{HWP}^{(2)}}$

This dependence of the number of coincidence counts in the state $| DD\rangle $ with the tilt angle *φ* can be seen in Fig. 10. For angles $\varphi = -22^{\circ}$, −1^∘^ and 19^∘^, the relative phase between components is equal to $\phi ' = -2\pi $, 0 and 2*π* while for $\varphi = -15^{\circ}$ and 12^∘^, the relative phase between components is equal to $\phi ' = -\pi $ and *π*.

Here we use a tilted HWP for walk-off compensation between photons downconverted in the first and the second BBO crystal. Should be noted that in other works and commercial devices it also common the use of compensation crystals, such as YVO, see Ref. [24, 33].

## Entanglement characterization, quantum state tomography and Bell test

Once the setup is optimally aligned, we can characterize and perform a Bell test in all four Bell states. As explained above, the evaluation of the CHSH inequality requires measuring several correlation functions, Eq. (13), which contain coincidence probabilities among the detectors. Thus, to start characterizing the correlations arising in our detectors, we compare quantum mechanical predictions to our data for two such correlations. In particular, we concentrate on $C(0^{\circ},\theta )$ and $C(45^{\circ},\theta )$ defined as, see Appendix C,

23 $$\begin{aligned} C(0^{\circ},\theta ) &= |\langle \Psi | V_{0}V_{\theta}\rangle |^{2}, \\ C(45^{\circ},\theta ) &=|\langle \Psi | V_{45^{\circ}}V_{\theta} \rangle |^{2} . \end{aligned}$$

These can be experimentally obtained by measuring the coincidence counts of pairs of photons in the state $| V_{0^{\circ}}\rangle \otimes | V_{\theta}\rangle $ or $| V_{45^{\circ}}\rangle \otimes | V_{\theta}\rangle $ respectively, while varying the angle *θ* at which we measure the state of the second photon.

**Figure 11:**
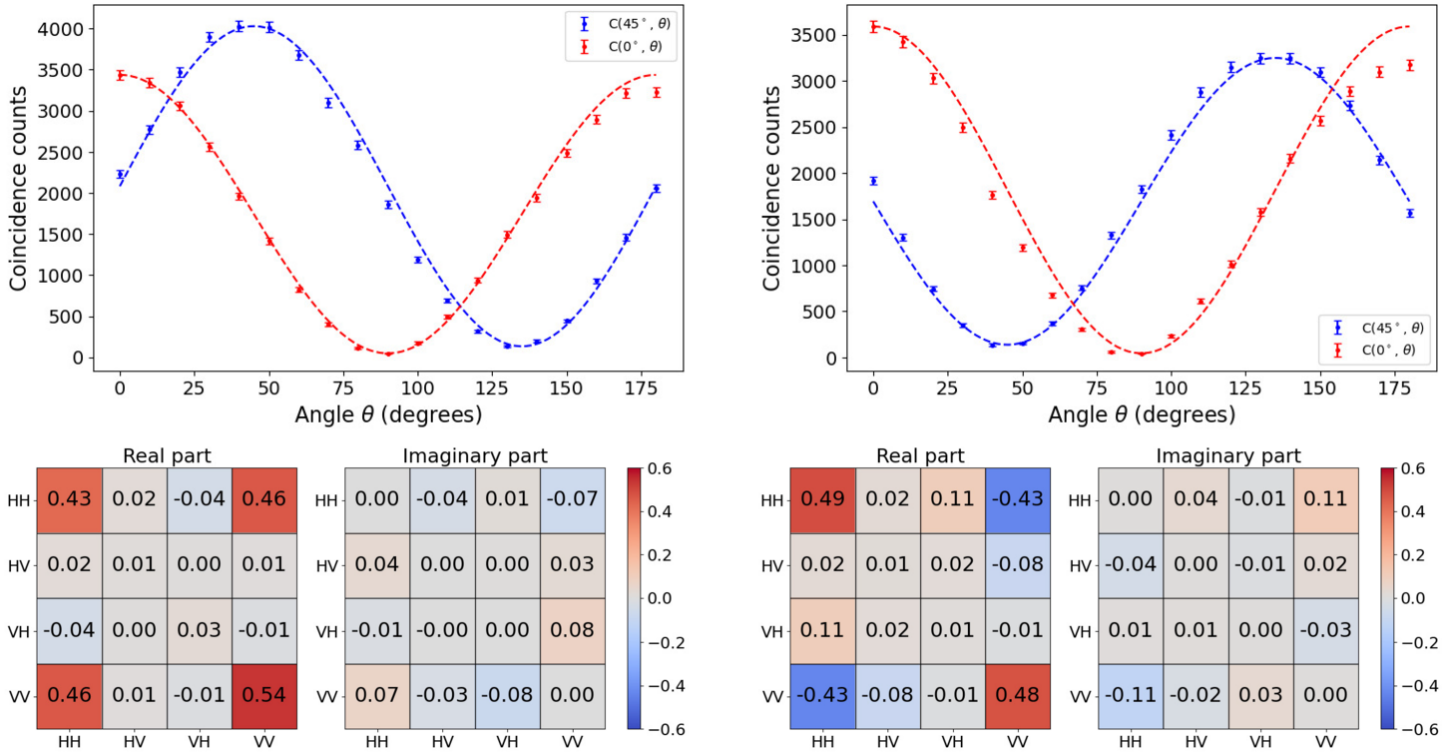
Unnormalized $C(0^{\circ},\theta )$, $C(45^{\circ},\theta )$ functions and tomography for the states $| \Phi ^{+}\rangle $ (left) and $| \Phi ^{-}\rangle $ (right). The dashed lines correspond to the quantum mechanics predictions, $C(\theta _{1},\theta _{2}) \propto \cos ^{2}(\theta _{1}-\theta _{2})$ and $C(\theta _{1},\theta _{2}) \propto \cos ^{2}(\theta _{1}+\theta _{2})$, for $| \Phi ^{+}\rangle $ and $| \Phi ^{-}\rangle $, respectively. The explicit expressions are derived in Appendix C

**Figure 12:**
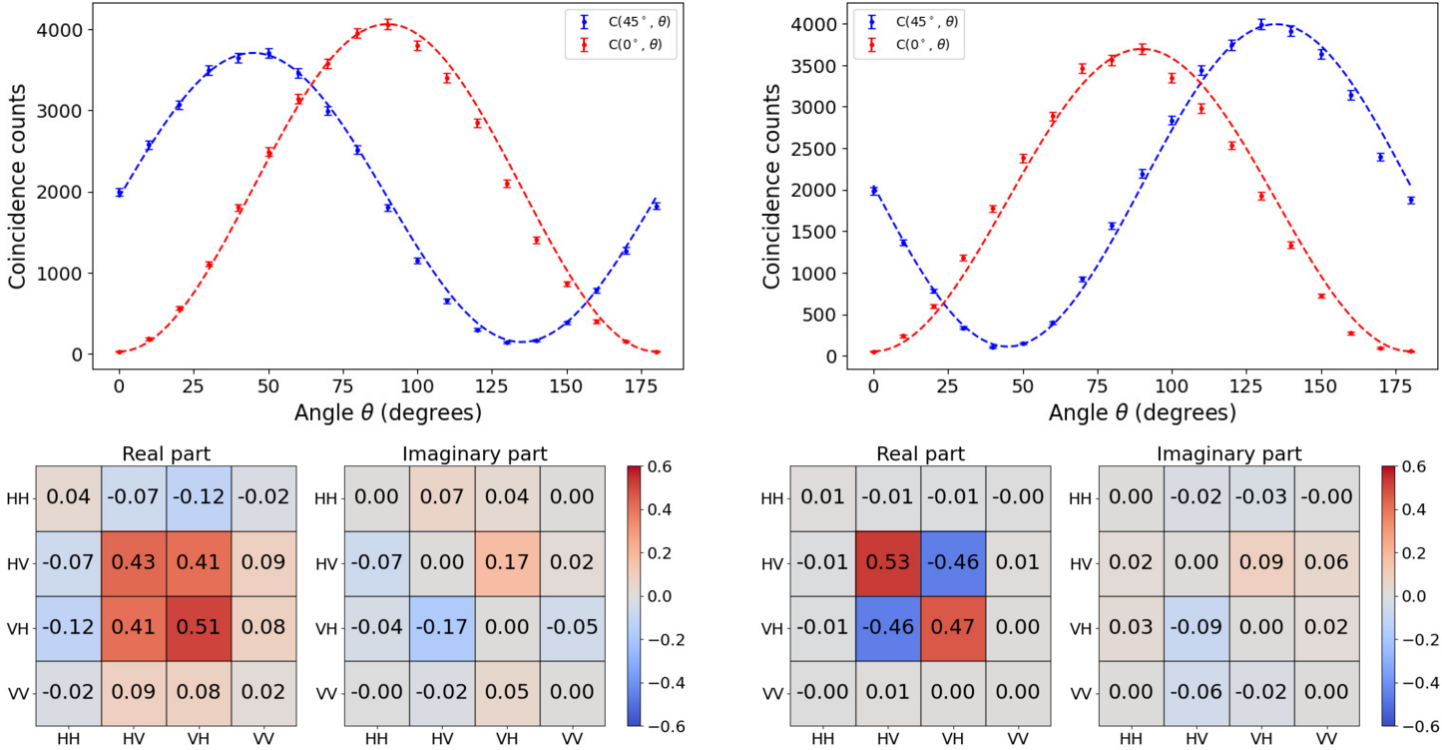
Unnormalized $C(0^{\circ},\theta )$, $C(45^{\circ},\theta )$ graphs and tomography for the states $| \Psi ^{+}\rangle $ (left) and $| \Psi ^{-}\rangle $ (right). The dashed lines correspond to the quantum mechanics predictions, $C(\theta _{1},\theta _{2}) \propto \sin ^{2}(\theta _{1}+\theta _{2})$ and $C(\theta _{1},\theta _{2}) \propto \sin ^{2}(\theta _{1}-\theta _{2})$, for $| \Psi ^{+}\rangle $ and $| \Psi ^{-}\rangle $, respectively. The explicit expressions are derived in Appendix C

Our measurements, compared to the quantum mechanical predictions are presented in the upper panels of Figs. 11 and 12. In all cases, the agreement between the experimental measurements, symbols, and the theoretical predictions, dashed lines, is very good. Note we are reporting results for the four different Bell states presented in Eq. (12). The details of the quantum mechanical predictions are provided in Appendix C.

### Quantum state tomography

Importantly, our setups allow also to perform a full quantum state tomography of the two-photon wave functions. In this way, we can quantify to which extent we are able to produce the desired quantum states. The density matrix of our two photon state is a $4\times 4$ complex matrix, see Eq. (5). As explained above it can be rewritten as products of Pauli operators, see Eq. (6). The latter form is easier to handle as its Stoke’s coefficients can be directly related to our measurements as explicitly provided in Eq. (B.2).

Our experimental results for the tomography of the four Bell states are presented in the colored charts in Figs. 11 and 12. In all cases we present the real, left square, and imaginary part, right square, of the corresponding density matrix. The theoretical predictions are easy to obtain. As an example, for instance in the $| \Phi ^{-}\rangle $ case we have,

24 $$\begin{aligned} \hat{\rho} &= | \Phi ^{-}\rangle \langle \Phi ^{-}| \\ &= {\frac{1}{\sqrt{2}}} (| HH\rangle - | VV\rangle ) {\frac{1}{\sqrt{2}}} ( \langle HH| - \langle VV|) \\ &= {\frac{1}{2}} \left ( | HH\rangle \langle HH| - | HH\rangle \langle VV| \right . \\ & - \left . | VV\rangle \langle HH| + | VV\rangle \langle VV| \right ) . \end{aligned}$$

In our two-photon basis, ($\{|HH\rangle , |HV\rangle , |VH\rangle , |VV \rangle \}$), this translates into the matrix,

25 $$ \hat{\rho} = \begin{bmatrix} {\frac{1}{2}} & 0 & 0 & -{\frac{1}{2}} \\ 0 & 0 & 0 & 0 \\ 0 & 0 & 0 & 0 \\ -{\frac{1}{2}} & 0 & 0 & {\frac{1}{2}} \end{bmatrix} . $$

This matrix should be compared to the entries in the right part of Fig. 11. First, we note that the imaginary part of the matrix elements, which should be zero according to the theoretical prediction, is found in most cases below 0.05, with two values reaching 0.11 in absolute value. The real parts show also a very good agreement with the theory predictions, with the two external diagonal entries being 0.49 and 0.48, and the antidiagonal ones, −0.43 and −0.43. The distance between the measured two-photon state and the theoretically expected one is provided by the fidelity, Eq. (9), reported in Table 4.

**Table 4:** Bell test and fidelity results for each of states

State	〈*S*〉	$\langle S' \rangle $	Fidelity
$| \Phi ^{+}\rangle $	2.765 ± 0.018	0.022 ± 0.018	0.945 ± 0.005
$| \Phi ^{-}\rangle $	0.101 ± 0.016	2.745 ± 0.016	0.918 ± 0.005
$| \Psi ^{+}\rangle $	−0.055 ± 0.019	−2.806 ± 0.019	0.881 ± 0.005
$| \Psi ^{-}\rangle $	−2.804 ± 0.018	−0.053 ± 0.018	0.954 ± 0.005

The discussion of the other Bell states is in general trends similar. We obtain imaginary parts which are fairly small, and values close to ±0.5 in the corresponding entries of the real part. In all cases, the fidelity obtained is above 0.88, see all values compiled in Table 4.

### Bell test

Finally, to prove that we have indeed an entangled state, we perform the Bell test. For each of the states, we obtain a violation of the Bell inequalities with at least 40 standard deviations from the maximum classical value of $| \langle S \rangle | = 2$, see Table 4. As each measurement takes 30 seconds, the time needed for conducting a Bell test using Setup 1 is 20 minutes and 5 minutes with Setup 2. The resulting value for the state $| \Phi ^{+}\rangle $ is $\langle S \rangle = 2.730 \pm 0.015$.

For the tomography and the calculation of the fidelity, we use only the number of coincidence counts, while for obtaining the value of the Bell inequality, we use the difference between coincidence counts and accidental counts, as described in Sect. D. Eliminating the accidental counts from the total coincidences allows us to suppress the contribution of background noise and the detectors’ dark counts. Together with a precise preparation of the entangled state, this leads to CHSH inequality values very close to the theoretical ones.

## Conclusions

We have presented a new experimental laboratory aimed at the undergraduate and master level to study quantum entangled photons. This laboratory is based on the method of obtaining entangled photons in two type I BBO crystals, first proposed and experimentally realized in Ref [30] and developed for the undergraduate laboratory in Refs. [18, 19] and later in Refs. [20, 21].

Two different setups have been described, which differ on the photon detection part. The photon detection is either performed with two single photon detectors or with four, allowing in the latter case to reduce the measurement time by a factor four.

The photons are collected by means of optical fibers mounted on custom made rails, thus ensuring an easy and robust alignment. The procedure to assemble and align the system from scratch, which has proven key in our experience, has been presented, thus providing a direct guide to future undergrad students in quantum science and technology laboratories worldwide.

The experiments which can be conducted are manifold. First, one can produce any of the well know Bell states, in our case produced with a fidelity higher that 88%. The full tomography of the states can be performed, thus confirming that the desired two-photon quantum state has been produced. Besides, one can also perform correlated measurements within the two photons, which can be directly confronted with quantum mechanical predictions.

Bell tests tailored for the different Bell states can also be performed. In our case, we measured violations of the corresponding inequalities by more than 40 standard deviations. With this setup, after alignment, a Bell test can be conducted in less than an hour.

All of this, combined with the fact that the total cost of components required to assemble both setups is approximately twenty thousand euros (see Sect. A for our total cost estimation and Refs. [19, 21] for other cost estimations of previous works), makes this experiment accessible to laboratories with limited resources. It also brings these type of demonstrations closer to undergraduate students, high school students, or even a broader audience. This facilitates the dissemination of key concepts in quantum mechanics beyond universities and specialized research groups.

## Figures and Tables

**Figure 13:**
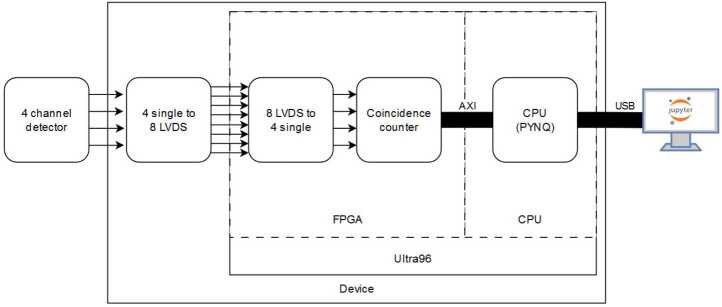
Block diagram of the coincidence counting system

**Figure 14:**
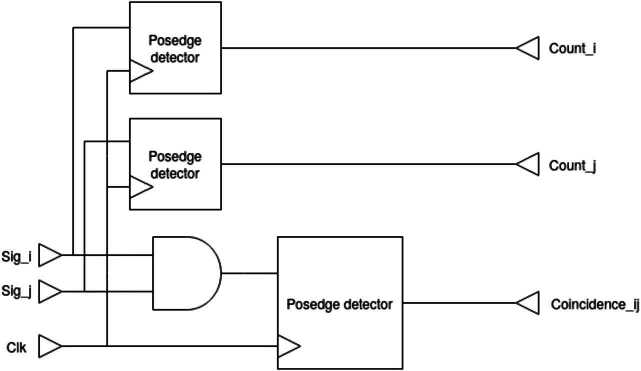
Logic involved in the counting and computation of the coincidences

**Figure 15:**
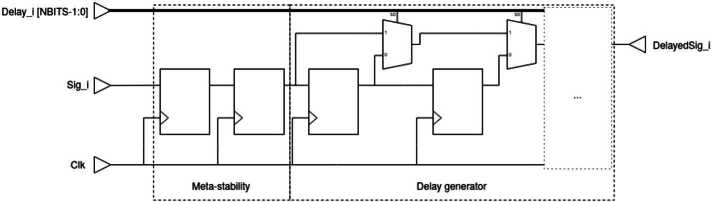
Logic involved in the delay generator with the non-optional registers to prevent meta-stability issues

## Data Availability

Data is provided within the manuscript.

## Appendix A: List of material

For any reader interested in replicating either of the two setups, here, in Table 5, there is an inventory of useful information for acquiring all the necessary elements. The total cost of all these elements amounts to around 20.000 euros. Also, more information about the total cost of similar experiments can be found in Refs. [19, 21].

**Table 5:** Detailed equipment used in the setups. The subscripts 1 and 2 denote the quantities of elements required solely for the construction of setups 1 and 2, respectively

Description of the product	Reference and Company	Price (eur.)	Quantity
Precision kinematic mount	KS1, ThorLabs	90.51	4 + 2_2_
Fiber-coupling lenses	F810FC-780, ThorLabs	267.73	2 + 2_2_
Goniometer	RP01/M, ThorLabs	101.24	1
Rotation Mount	RSP1X225/M, ThorLabs	141.46	4
Rotation Mount 30 mm cage system	CRM1T/M, ThorLabs	87.20	1
BBO crystals mount	KS1RS, ThorLabs	250.90	1
PBS mount	KM200PM/M, ThorLabs	127.41	2_2_
4-channel detector	SPCM-AQ4C, Excelitas Technologies	13,456.21	1
Current and Temperature Controllers for Laser Diodes	LTC56A/M, ThorLabs	2847.77	1
Laser diode 404 nm 400 mW	L404P400M, ThorLabs	684.60	1
Mirrors	BB1-E02, ThorLabs	73.83	2
BBO crystals	EKSMA Optics	1540.00	1
Bandpass Filter 405 nm	FBH405-10, ThorLabs	149.69	1
Bandpass Filter 800 nm	FBH800-40, ThorLabs	149.69	2 + 2_2_
“Infrared” Polarizers	LPNIRE100-B, ThorLabs	116.46	2_1_
Quarter-wave Plate 808 nm	WPQ05M-808, ThorLabs	461.09	2
Half-wave Plate 808 nm	WPH05M-808, ThorLabs	461.09	2_2_
Half-wave Plate 405 nm	WPH05M-405, ThorLabs	461.09	2
Polarizing Beamsplitter (PBS)	PBS252, ThorLabs	235.40	2_2_
Development board for the coincidence circuit	NUCLEO-F756ZG, STMicroelectronics	22.82	1

## Appendix B: Stokes coefficients

The general equation for each of the Stokes coefficients of Eq. (6) is

B.1 $$ S_{ij} = \mathrm{Tr}(\hat{\sigma _{i}} \otimes \hat{\sigma _{j}} \cdot \hat{\rho}), $$

where *ρ̂* is the state of our pair of photons. Thus, the explicit expression for the Stokes coefficients is,

$$\begin{aligned} &S_{00} = P_{| HH\rangle } + P_{| HV\rangle } + P_{| VH\rangle } + P_{| VV\rangle }, \\ &S_{01} = P_{| HD\rangle } - P_{| HA\rangle } + P_{| VD\rangle } - P_{| VA\rangle }, \\ &S_{02} = P_{| HL\rangle } - P_{| HR\rangle } + P_{| VL\rangle } - P_{| VR\rangle }, \\ &S_{03} = P_{| HH\rangle } - P_{| HV\rangle } + P_{| VH\rangle } - P_{| VV\rangle }, \\ &S_{10} = P_{| DH\rangle } + P_{| DV\rangle } - P_{| AH\rangle } - P_{| AV\rangle }, \\ &S_{11} = P_{| DD\rangle } - P_{| DA\rangle } - P_{| AD\rangle } + P_{| AA\rangle }, \\ &S_{12} = P_{| DL\rangle } - P_{| DR\rangle } - P_{| AL\rangle } + P_{| AR\rangle }, \\ &S_{13} = P_{| DH\rangle } - P_{| DV\rangle } - P_{| AH\rangle } + P_{| AV\rangle }, \\ &S_{20} = P_{| LH\rangle } + P_{| LV\rangle } - P_{| RH\rangle } - P_{| RV\rangle }, \\ &S_{21} = P_{| LD\rangle } - P_{| LA\rangle } - P_{| RD\rangle } + P_{| RA\rangle }, \\ &S_{22} = P_{| LL\rangle } - P_{| LR\rangle } - P_{| RL\rangle } + P_{| RR\rangle }, \\ &S_{23} = P_{| LH\rangle } - P_{| LV\rangle } - P_{| RH\rangle } + P_{| RV\rangle }, \\ &S_{30} = P_{| HH\rangle } + P_{| HV\rangle } - P_{| VH\rangle } - P_{| VV\rangle }, \\ &S_{31} = P_{| HD\rangle } - P_{| HA\rangle } - P_{| VD\rangle } + P_{| VA\rangle }, \\ &S_{32} = P_{| HL\rangle } - P_{| HR\rangle } - P_{| VL\rangle } + P_{| VR\rangle }, \\ &S_{33} = P_{| HH\rangle } - P_{| HV\rangle } - P_{| VH\rangle } + P_{| VV\rangle }. \end{aligned}$$

Using Eq. (6), we can write the reconstructed density matrix and as we can see in Eq. (B.2). This matrix is hermitian ($\hat{\rho} = \hat{\rho}^{\dagger}$) and normalized ($\mathrm{Tr}( \hat{\rho}) = 1$) by definition.

B.2 $$ \hat{\rho} = \frac{1}{4} \begin{pmatrix} S_{00} + S_{03} + S_{30} + S_{33} & S_{01} + S_{31} - i (S_{02} + S_{32}) & S_{10} + S_{13} - i (S_{20} + S_{23}) & S_{11} - S_{22} - i (S_{12} + S_{21}) \\ S_{01} + S_{31} + i (S_{02} + S_{32}) & S_{00} - S_{03} + S_{30} - S_{33} & S_{11} - S_{22} + i (S_{12} - S_{21}) & S_{10} - S_{13} - i (S_{20} - S_{23}) \\ S_{10} + S_{13} + i (S_{20} + S_{23}) & S_{11} - S_{22} - i (S_{12} - S_{21}) & S_{00} + S_{03} - S_{30} - S_{33} & S_{01} - S_{31} - i (S_{02} - S_{32}) \\ S_{11} - S_{22} + i (S_{12} + S_{21}) & S_{10} - S_{13} + i (S_{20} - S_{23}) & S_{01} - S_{31} + i (S_{02} - S_{32}) & S_{00} - S_{03} - S_{30} + S_{33} \end{pmatrix} . $$

The matrix presented in Eq. (B.2) is the one we have used for obtaining the colored charts in Fig. 11 and 12. In these representations, the weights that accompany the real (imaginary) part for each one of the entries of this matrix are displayed with different colors depending on their relative weight with respect to the other coefficients in the “Real part” (“Imaginary part”) chart.

## Appendix C: Computation of $P_{| V_{\theta _{1}}V_{\theta _{2}}\rangle }$ for all four Bell states

We define $P_{| V_{\theta _{1}}V_{\theta _{2}}\rangle }$ as the probability of finding the two photons in the state $| V_{\theta _{1}}\rangle \otimes | V_{\theta _{2}}\rangle $. The values of this probabilities for each one of the states are:

- For $| \Phi ^{+}\rangle = \frac{1}{\sqrt{2}} (| HH\rangle + | VV\rangle )$:
  C.1 $$\begin{aligned} P_{| V_{\theta _{1}}V_{\theta _{2}}\rangle } &= |\langle \Phi ^{+} | V_{ \theta _{1}}V_{\theta _{2}} \rangle |^{2} \\ &= |\frac{1}{\sqrt{2}} (\langle HH| + \langle VV|) \cdot (\sin \theta _{1} \sin \theta _{2} | HH\rangle + \cos \theta _{1} \cos \theta _{2} | VV\rangle \\ &\quad{} -\sin \theta _{1} \cos \theta _{2} | HV\rangle -\cos \theta _{1} \sin \theta _{2} | VH\rangle )|^{2} \\ &= |\frac{1}{\sqrt{2}} (\sin \theta _{1} \sin \theta _{2} + \cos \theta _{1} \cos \theta _{2})|^{2} \\ & = |\frac{1}{\sqrt{2}} \cos(\theta _{1} - \theta _{2})|^{2} = \frac{1}{2} \cos ^{2}(\theta _{1} - \theta _{2}) \end{aligned}$$
- For $| \Phi ^{-}\rangle = \frac{1}{\sqrt{2}} (| HH\rangle - | VV\rangle )$:
  C.2 $$\begin{aligned} P_{| V_{\theta _{1}}V_{\theta _{2}}\rangle } &= |\langle \Phi ^{-} | V_{ \theta _{1}}V_{\theta _{2}} \rangle |^{2} \\ &= |\frac{1}{\sqrt{2}} (\langle HH| - \langle VV|) \cdot (\sin \theta _{1} \sin \theta _{2} | HH\rangle + \cos \theta _{1} \cos \theta _{2} | VV\rangle \\ &\quad{} -\sin \theta _{1} \cos \theta _{2} | HV\rangle -\cos \theta _{1} \sin \theta _{2} | VH\rangle )|^{2} \\ &= |\frac{1}{\sqrt{2}} (\sin \theta _{1} \sin \theta _{2} - \cos \theta _{1} \cos \theta _{2})|^{2} \\ &\quad = |\frac{1}{\sqrt{2}} \cos(\theta _{1} + \theta _{2})|^{2} = \frac{1}{2} \cos ^{2}(\theta _{1} + \theta _{2}) \end{aligned}$$
- For $| \Psi ^{+}\rangle = \frac{1}{\sqrt{2}} (| HV\rangle + | VH\rangle )$:
  C.3 $$\begin{aligned} P_{| V_{\theta _{1}}V_{\theta _{2}}\rangle } &= |\langle \Psi ^{+} | V_{ \theta _{1}}V_{\theta _{2}} \rangle |^{2} \\ &= |\frac{1}{\sqrt{2}} (\langle HV| + \langle VH|) \cdot (\sin \theta _{1} \sin \theta _{2} | HH\rangle + \cos \theta _{1} \cos \theta _{2} | VV\rangle \\ &\quad{} -\sin \theta _{1} \cos \theta _{2} | HV\rangle -\cos \theta _{1} \sin \theta _{2} | VH\rangle )|^{2} \\ &= |\frac{1}{\sqrt{2}} (\sin \theta _{1} \cos \theta _{2} + \cos \theta _{1} \sin \theta _{2})|^{2} \\ & = |\frac{1}{\sqrt{2}} \sin(\theta _{1} + \theta _{2})|^{2} = \frac{1}{2} \sin ^{2}(\theta _{1} + \theta _{2}) \end{aligned}$$
- For $| \Psi ^{-}\rangle = \frac{1}{\sqrt{2}} (| HV\rangle - | VH\rangle )$:
  C.4 $$\begin{aligned} P_{| V_{\theta _{1}}V_{\theta _{2}}\rangle } &= |\langle \Psi ^{-} | V_{ \theta _{1}}V_{\theta _{2}} \rangle |^{2} \\ &= |\frac{1}{\sqrt{2}} (\langle HV| - \langle VH|) \cdot (\sin \theta _{1} \sin \theta _{2} | HH\rangle + \cos \theta _{1} \cos \theta _{2} | VV\rangle \\ &\quad{} -\sin \theta _{1} \cos \theta _{2} | HV\rangle -\cos \theta _{1} \sin \theta _{2} | VH\rangle )|^{2} \\ &= |\frac{1}{\sqrt{2}} (\sin \theta _{1} \cos \theta _{2} - \cos \theta _{1} \sin \theta _{2})|^{2} \\ & = |\frac{1}{\sqrt{2}} \sin(\theta _{1} - \theta _{2})|^{2} = \frac{1}{2} \sin ^{2}(\theta _{1} - \theta _{2}) \end{aligned}$$

These theory predictions are compiles in Table 6.

**Table 6:** Values of $P_{| V_{\theta _{1}}V_{\theta _{2}}\rangle }$ for each one of the Bell states

Bell state	$P_{| V_{\theta _{1}}V_{\theta _{2}}\rangle }$
$| \Phi ^{+}\rangle $	$\frac{1}{2} \cos ^{2}(\theta _{1} - \theta _{2})$
$| \Phi ^{-}\rangle $	$\frac{1}{2} \cos ^{2}(\theta _{1} + \theta _{2})$
$| \Psi ^{+}\rangle $	$\frac{1}{2} \sin ^{2}(\theta _{1} + \theta _{2})$
$| \Psi ^{-}\rangle $	$\frac{1}{2} \sin ^{2}(\theta _{1} - \theta _{2})$

## Appendix D: Experimental data and error estimates

For completeness we provide the raw data and details on the way the different probabilities are estimated from the data including their error estimates.

### D.1 Experimental data

We provide all the data obtained for each one of the Bell test performed to the different Bell states using the setup depicted in Fig. 3, these results are collected in Tables 7, 8, 9 and 10. We also provide the data for the Bell test achieved with the photons in the state $| \Phi ^{+}\rangle $ in Table 11, using the setup presented in Fig. 4. In all these graphs, the angles *α* and *β* stands for the angles at which Alice ($| V_{\alpha}\rangle $) and Bob ($| V_{\beta}\rangle $) measure their respective photons. Each of these measurements was taken using a time interval of 30 seconds.

**Table 7:** Data obtained for the computation of the Bell inequality using pairs of photons in the $| \Phi ^{+}\rangle $

*α* (^∘^)	*β* (^∘^)	$N_{a}$	$N_{b}$	$N_{c}$	$N_{acc}$
45	22.5	242,324	126,944	3025	92.28
45	−22.5	241,250	125,920	377	91.13
45	−67.5	244,869	142,160	1242	104.43
45	−112.5	250,568	145,332	3959	109.25
0	22.5	231,257	137,000	3432	95.05
0	−22.5	235,528	132,632	2975	93.72
0	−67.5	226,909	138,908	310	94.56
0	−112.5	226,724	143,352	1028	97.50
−45	22.5	234,822	135,900	763	95.74
−45	−22.5	234,676	132,592	3684	93.35
−45	−67.5	233,508	145,184	3376	101.70
−45	−112.5	229,828	150,008	545	103.43
−90	22.5	224,928	129,820	394	87.60
−90	−22.5	222,538	123,984	927	82.77
−90	−67.5	217,928	135,868	3728	88.83
−90	−112.5	223,545	141,888	3031	95.16

**Table 8:** Data obtained for the computation of the Bell inequality using pairs of photons in the $| \Phi ^{-}\rangle $ state

*α* (^∘^)	*β* (^∘^)	$N_{a}$	$N_{b}$	$N_{c}$	$N_{acc}$
45	22.5	220,634	157,440	721	104.21
45	−22.5	226,207	151,083	3995	102.53
45	−67.5	226,708	173,348	4562	117.90
45	−112.5	221,408	182,304	1134	121.09
0	22.5	208,526	157,300	4001	98.40
0	−22.5	214,617	149,412	3915	96.20
0	−67.5	208,804	169,832	565	106.38
0	−112.5	213,806	182,984	1001	117.37
−45	22.5	245,625	157,568	4293	116.11
−45	−22.5	246,193	149,052	683	110.09
−45	−67.5	240,321	168,744	1417	121.66
−45	−112.5	241,023	178,476	4720	129.05
−90	22.5	259,133	157,176	852	122.19
−90	−22.5	259,584	149,428	835	116.37
−90	−67.5	254,570	174,292	4523	133.11
−90	−112.5	259,205	182,424	4554	141.86

**Table 9:** Data obtained for the computation of the Bell inequality using pairs of photons in the $| \Psi ^{+}\rangle $ state

*α* (^∘^)	*β* (^∘^)	$N_{a}$	$N_{b}$	$N_{c}$	$N_{acc}$
45	22.5	202,953	140,256	2997	85.40
45	−22.5	209,729	140,292	589	88.27
45	−67.5	199,521	132,980	854	79.60
45	−112.5	199,216	138,172	3409	82.58
0	22.5	195,788	135,044	520	79.32
0	−22.5	191,147	126,572	716	72.58
0	−67.5	196,727	130,908	3435	77.26
0	−112.5	191,245	138,028	3167	79.19
−45	22.5	185,983	135,516	648	75.61
−45	−22.5	187,608	130,676	3189	73.55
−45	−67.5	188,891	133,528	2898	75.67
−45	−112.5	185,914	136,744	470	76.27
−90	22.5	179,726	137,788	2973	74.29
−90	−22.5	178,394	131,576	2709	70.42
−90	−67.5	179,378	131,052	401	70.52
−90	−112.5	175,836	133,828	642	70.60

**Table 10:** Data obtained for the computation of the Bell inequality using pairs of photons in the $| \Psi ^{-}\rangle $ state

*α* (^∘^)	*β* (^∘^)	$N_{a}$	$N_{b}$	$N_{c}$	$N_{acc}$
45	22.5	205,843	152,544	1026	94.20
45	−22.5	202,783	146,180	3625	88.93
45	−67.5	203,926	136,128	2977	83.28
45	−112.5	203,209	140,200	370	85.47
0	22.5	199,956	155,508	476	93.28
0	−22.5	198,214	149,576	728	88.94
0	−67.5	194,361	139,668	3686	81.44
0	−112.5	195,118	143,816	3027	84.18
−45	22.5	209,593	155,160	3400	97.56
−45	−22.5	209,790	149,908	643	94.35
−45	−67.5	207,594	136,140	740	84.79
−45	−112.5	202,074	142,580	3403	86.44
−90	22.5	197,175	147,948	3358	87.51
−90	−22.5	202,596	146,072	3212	88.78
−90	−67.5	204,167	135,736	438	83.14
−90	−112.5	202,654	143,952	860	87.52

**Table 11:** Data obtained for the computation of the Bell inequality with the setup depicted in Fig. 4, using pairs of photons in the $| \Phi ^{+}\rangle $ state

*α* (^∘^)	*β* (^∘^)	$N_{a}$	$N_{b}$	$N_{c}$	$N_{acc}$
45	22.5	305,415	248,380	5302	227.57
45	−22.5	280,234	237,452	1709	199.62
45	−67.5	190,449	247,220	859	141.24
45	−112.5	210,298	247,104	5626	155.89
0	22.5	305,380	320,864	6682	293.95
0	−22.5	288,215	312,288	6478	270.01
0	−67.5	194,873	321,212	1267	187.78
0	−112.5	201,912	301,124	1538	182.40
−45	22.5	294,989	231,004	2013	204.43
−45	−22.5	280,329	230,040	5343	193.46
−45	−67.5	184,691	232,300	4809	128.71
−45	−112.5	202,687	237,160	604	144.20
−90	22.5	292,146	164,976	729	144.59
−90	−22.5	278,696	170,320	513	142.40
−90	−67.5	174,801	158,404	4295	83.06
−90	−112.5	201,492	164,912	4770	99.68

### D.2 Probability estimates and error analysis

Assuming that the counts follow a Poisson distribution, the error corresponding to the number of counts detected in a certain time interval is equal to the square root of that value. That is:

D.1 $$ \sigma _{N_{a}} = \sqrt{N_{a}}\,, \qquad \sigma _{N_{b}} = \sqrt{N_{b}} \,, \qquad \sigma _{N_{c}} = \sqrt{N_{c}}. $$

Therefore, performing error propagation in Eq. (22), we have that the error in $N_{acc}$ is,

D.2 $$ \sigma _{N_{acc}} = \frac{\tau}{T}\cdot \sqrt{N_{b}^{2} \cdot \sigma _{N_{a}}^{2} + N_{a}^{2} \cdot \sigma _{N_{b}}^{2}} = \frac{\tau}{T}\cdot \sqrt{N_{b}^{2} \cdot N_{a} + N_{a}^{2} \cdot N_{b}}, $$

where we have assumed that the values *T* and *τ* have no associated error. For simplicity, we define $N_{VV} = N_{| V_{\alpha }V_{\beta}\rangle } = N_{c}( | V_{\alpha }V_{\beta}\rangle ) - N_{acc}(| V_{\alpha }V_{\beta}\rangle )$ as the number of detected coincidence counts minus the number of accidental coincidence counts and $\sigma _{N_{VV}} = \sigma _{N}(| V_{\alpha }V_{\beta}\rangle )$ to their associated error,

D.3 $$ \sigma _{N_{VV}} = \sqrt{\sigma ^{2}_{N_{c}}( | V_{\alpha }V_{\beta}\rangle ) + \sigma ^{2}_{N_{acc}}( | V_{\alpha }V_{\beta}\rangle )}. $$

Then, the equation for the different probabilities is

D.4 $$ \begin{aligned} P_{| V_{\alpha }V_{\beta}\rangle } &= \frac{N_{| V_{\alpha }V_{\beta}\rangle }}{N_{| V_{\alpha }V_{\beta}\rangle } + N_{| V_{\alpha -90^{\circ}} V_{\beta}\rangle } + N_{| V_{\alpha }V_{\beta -90^{\circ}}\rangle } + N_{| V_{\alpha -90^{\circ}} V_{\beta -90^{\circ}}\rangle }} \\ &= \frac{N_{| V_{\alpha }V_{\beta}\rangle }}{N_{| V_{\alpha }V_{\beta}\rangle } + N_{| H_{\alpha} V_{\beta}\rangle } + N_{| V_{\alpha }H_{\beta}\rangle } + N_{| H_{\alpha} H_{\beta}\rangle }} \\ &= \frac{N_{VV}}{N_{VV} + N_{HV} + N_{VH} + N_{HH}}, \end{aligned} $$

where, from the Eq. (4), we extract that $| V_{\gamma -90^{\circ}}\rangle = | H_{\gamma}\rangle $. The error associated to the probability $P_{| V_{\alpha }V_{\beta}\rangle }$ can be written as

D.5 $$ \begin{aligned}[b] \sigma _{P_{| V_{\alpha }V_{\beta}\rangle }} &= \frac{1}{\left ( N_{VV} + N_{HH} + N_{VH} + N_{HH} \right )^{2}} \\ &\quad{} \cdot \sqrt{ N^{2}_{VV} \cdot (\sigma _{N_{VH}}^{2} + \sigma _{N_{HV}}^{2} + \sigma _{N_{HH}}^{2} ) + (N_{HV} + N_{VH} + N_{HH})^{2} \cdot \sigma _{N_{VV}}^{2} }. \end{aligned} $$

For the correlation functions, defined as in Eq. (13), we have that the error is

D.6 $$ \begin{aligned} \sigma _{E(\alpha , \beta )} = \sqrt{\sigma ^{2}_{P_{ | V_{\alpha }V_{\beta}\rangle }} + \sigma ^{2}_{P_{ | V_{\alpha }H_{\beta}\rangle }} + \sigma ^{2}_{P_{ | H_{\alpha }V_{\beta}\rangle }} + \sigma ^{2}_{P_{ | H_{\alpha }H_{\beta}\rangle }}}. \end{aligned} $$

Finally, for both functions *S* and $S'$, defined in Eq. (14) and Eq. (15), we have the same associated error,

D.7 $$ \sigma _{S} = \sigma _{S'} = \sqrt{\sigma ^{2}_{E(\alpha , \beta )} + \sigma ^{2}_{E(\alpha , \beta ')} + \sigma ^{2}_{E(\alpha ', \beta )} + \sigma ^{2}_{E(\alpha ', \beta ')}}. $$

Also, we define the coverage ratio as the distance in standard deviations between the absolute value of our value obtained in the CHSH inequality and the maximum value that can be obtained clasically, i.e. 2. Thus,

D.8 $$ \begin{aligned} \mathrm{{Coverage~Ratio} }&= \frac{|\langle S \rangle | - 2}{\sigma _{S}} \quad\text{for } | \Phi ^{+}\rangle \quad\text{and}\quad | \Psi ^{-}\rangle \\ &= \frac{|\langle S' \rangle | - 2}{\sigma _{S'}} \quad\text{for } | \Phi ^{-}\rangle \quad\text{and}\quad | \Psi ^{+}\rangle . \end{aligned} $$

## Appendix E: Coincidence detection

In order to count and compute the coincidences among the four channels, an HDL module has been designed to be implemented in an FPGA. More specifically, the counter has been designed to work in an Ultra69-v1 board with the Zynq Ultrascale+ MPSoC. This allows for a very convenient setup that is simple to use and can be reproduced easily. Since its design is parameterizable, it can work with more than 4 input signals and the coincidence registers for every combination are automatically generated in synthesis (the process where the HDL code is converted into hardware).

This SoC is divided into two main parts: the CPU and the Programmable Logic. This allows a bare-bones Linux image with Python and some extra dependencies to be installed on the device (PYNQ), which can control and communicate with the PL through an AXI (Lite) bus. This way, when connecting the device to a computer either through USB or Ethernet (or even Wi-Fi), the user is encountered with a very user-friendly and intuitive Jupyter Notebook interface through which the whole acquisition is done. The block diagram describing the parts of the device is shown in Fig. 13.

Aside from the Ultra96-v1, a mezzanine board is added to convert the single-ended signals into LVDS signals (for better accuracy, compatibility, and protection), which are then reconverted into single-ended signals inside the PL.

In order to both count the pulses and compute the coincidences, the following structures run in parallel (which output their signals into a counter that increments the stored value by 1 every time its positive edge detector spikes), which can be seen in Fig. 14:

- A positive edge detector for every signal that is inputted
- An AND gate followed by a positive edge detector that spikes when a coincidence has occurred between the two selected channels (an overlap of the two signals is detected, but only when the overlap first happens; not the whole period the signals stay up).

Aside from that, a configurable delay generator has been added in order to be able to correct for different path lengths among the channels in the detection system. This module consists of a series of registers and multiplexers (as seen in Fig. 15) that allow you to choose how many delay registers you want to put on every channel. It’s worth noting that behind every input, two non-optional registers are stacked to correct and prevent meta-stability issues.
